# Production of Two Isomers of Sphaeralcic Acid in Hairy Roots from *Sphaeralcea angustifolia*

**DOI:** 10.3390/plants12051090

**Published:** 2023-03-01

**Authors:** Karen Barrera, Manasés González-Cortazar, Rogelio Reyes-Pérez, Dolores Pérez-García, Maribel Herrera-Ruiz, Jesús Arellano-García, Francisco Cruz-Sosa, Pilar Nicasio-Torres

**Affiliations:** 1 Departamento de Biotecnología, Universidad Autónoma Metropolitana-Iztapalapa (UAM-Iztapalapa), Av. Ferrocarril de San Rafael Atlixco 186, Col. Leyes de Reforma 1a. Sección, Iztapalapa, Ciudad de Mexico 09310, Ciudad de México, Mexico; 2 Centro de Investigación Biomédica del Sur (CIBIS), Instituto Mexicano del Seguro Social (IMSS), Argentina No. 1 Col. Centro, Xochitepec 62790, Morelos, Mexico; 3 Centro de Investigación en Biotecnología (CeIB), Universidad Autónoma del Estado de Morelos (UAEM), Circuito Universidad 1001, Cuernavaca 62209, Morelos, Mexico

**Keywords:** anti-inflammatory, Malvaceae, scopoletin, *Sphaeralcea angustifolia*, sphaeralcic acid, sphaeralcic acid isomers

## Abstract

The *Sphaeralcea angustifolia* plant is used as an anti-inflammatory and gastrointestinal protector in Mexican traditional medicine. The immunomodulatory and anti-inflammatory effects have been attributed to scopoletin (**1**), tomentin (**2**), and sphaeralcic acid (**3**) isolated from cells in suspension cultures and identified in the aerial tissues of the wild plant. The hairy roots from *S. angustifolia* established by infecting internodes with *Agrobacterium rhizogenes* were explored to produce active compounds based on biosynthetic stability and their capacity to produce new compounds. Chemical analysis was resumed after 3 years in these transformed roots, SaTRN12.2 (line 1) produced scopoletin (0.0022 mg g^−1^) and sphaeralcic acid (0.22 mg g^−1^); instead, the SaTRN7.1 (line 2) only produced sphaeralcic acid (3.07 mg g^−1^). The sphaeralcic acid content was 85-fold higher than that reported for the cells in the suspension cultivated into flakes, and it was similar when the cells in suspension were cultivated in a stirring tank under nitrate restriction. Moreover, both hairy root lines produced stigmasterol (**4**) and β-sitosterol (**5**), as well as two new naphthoic derivates: iso-sphaeralcic acid (**6**) and 8-methyl-iso-sphaeralcic acid (**7**), which turned out to be isomers of sphaeralcic acid (**3**) and have not been reported. The dichloromethane–methanol extract from SaTRN7.1 hairy root line had a gastroprotective effect on an ulcer model in mice induced with ethanol.

## 1. Introduction

*Sphaeralcea angustifolia* (Cavanilles) G. Don (Malvaceae) is used as an anti-inflammatory and to treat gastrointestinal diseases in Mexican traditional medicine [1,2,3]. The extracts from the aerial tissues of this plant exhibited an anti-inflammatory activity in acute (auricular edema induced with phorbol ester-TPA) and polyarthritis inflammation (induced with Freund’s complete adjuvant (FCA)) mouse models, with β-sitosterol, α- and β-amyrin, *trans*-cinnamic acid, and scopoletin identified as the main active compounds. Subsequently, using an FCA model, the extract decreased the serum levels of pro-inflammatory interleukin (IL)-1ß, IL-6, and tumor necrosis factor-alpha (TNF-α) and increased the anti-inflammatory IL-10 levels in the synovial fluid of mice with untreated polyarthritis [4,5,6]. With these backgrounds, a gel formulation containing 1% *S. angustifolia* dichloromethane extract standardized in the scopoletin content was evaluated in patients with osteoarthritis of the hands. The therapeutic effectiveness and tolerability of this phytomedicine were approximately 90% [7].

Scopoletin has been isolated from many plants [8] and important pharmacological activities have been reported, namely: anti-inflammatory [9,10]; antioxidant [11,12]; inhibition of nuclear transcription factor-kappa β (NF-κβ) and inflammatory cytokine production; inhibition of pro-inflammatory mediators [13,14]; anti-angiogenic in an arthritis mouse model [15]; anti-proliferative [16]; and gastroprotective as a potential preventive and therapeutic agent for gastro–esophageal inflammation, mainly through its anti-secretory and prokinetic activities [17].

**Figure 1:**
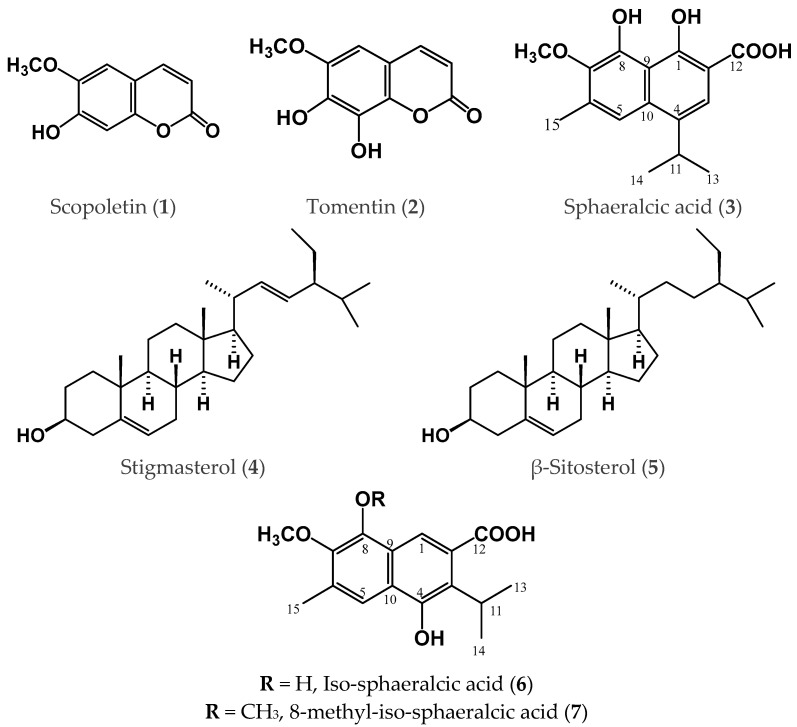
Compounds isolated from wild plants and cells in a suspension of *S. angustifolia* and identified in the dichloromethane–methanol extracts of the SaTRN12.2 (line 1) and SaTRN 7.1 (line 2) hairy root lines of *Sphaeralcea angustifolia*.

Cell suspension culture was employed as a feasible methodology to produce scopoletin, tomentin, and sphaeralcic acid (Figure 1); scopoletin and sphaeralcic acid production was improved by the total nitrate reduction in the culture medium [18,19,20]. The production of sphaeralcic acid in the cell suspension culture was scaled up using a bioreactor in a stirred tank and nitrate restriction [21]. Tomentin and sphaeralcic acid were identified as potent anti-inflammatories in acute (edema induced with λ-carrageenan footpad and with TPA) and chronic (kaolin/λ-carrageenan-induced arthritis) mice models [18,22,23]. Similarly, both compounds modulated the production of pro- (IL-1β) and anti-inflammatory (IL-10 and IL-4) cytokines [22,23].

Hairy root cultures from *S. angustifolia* were also established, mediated by node infection with *Agrobacterium rhizogenes* ATCC15834, to produce scopoletin and sphaeralcic acid [24]. The aim of this study was to characterize scopoletin and sphaeralcic acid production in the SaTRN12.2 and SaTRN7.1 hairy root lines from *S. angustifolia* after 3 years in culture. In addition, it aimed to determine the chemical profiles of dichloromethane–methanol extracts via the purification and identification of other secondary metabolites biosynthesized in these cultures; the sterols and sphaeralcic acid isomers were categorized as new. Moreover, we aimed to evaluate the anti-ulcerogenic effect of the dichloromethane–methanol extract of SaTRN7.1 hairy root line in the ulcer model in mice induced with ethanol; this extract comprised sphaeralcic acid and its two isomers, as well as scopoletin and β-sitosterol, which reported a gastro-protective effect.

## 2. Results and Discussion

### 2.1. S. angustifolia Hairy Root Cultures

#### Scopoletin and Sphaeralcic Acid Production

Hairy root culture is a useful biotechnological system for producing scopoletin and sphaeralcic acid in *S. angustifolia* [24]. The two hair root lines previously established and chemically analyzed in this project were classified according to the production of active compounds (Table 1): one, SaTRN12.2 (line 1), as a producer of two active compounds, (Figure 1) scopoletin (0.0022 mg g^−1^) and sphaeralcic acid (0.22 mg g^−1^), this hairy root line had been reported to be a not producer by Reyes-Pérez et al. 2022 [24]; a second one, SaTRN7.1 (line 2), as a strong producer of sphaeralcic acid (3.07 mg g^−1^) at 10-fold higher levels according to the analysis previously reported (scopoletin, 0.011 mg g^−1^ and sphaeralcic acid,1.22 mg g^−1^) for this hairy root line [24]. The sphaeralcic acid yield in the hairy root line 2 was 85-fold higher than that reported for the cells in the suspension (0.0359 mg g^−1^) of *S. angustifolia* cultivated in the MS medium with nitrate restriction [19], and similar to that reported in the same biotechnological system (3.47 mg g^−1^) cultivated in a stirring tank bioreactor [21].

### 2.2. Chemical Profiles of Dichloromethane–Methanol Extracts from S. angustifolia Hairy Roots Lines

#### 2.2.1. Stigmasterol and β-Sitosterol Identification

From the dichloromethane–methanol extract of SaTRN12.2 (line 1) of *S. angustifolia,* the thin-layer chromatography (TLC) analysis of sub-fraction SaL1F3R6 (12 mg) revealed the presence of sterols. The compounds stigmasterol (**4**) and β-sitosterol (**5**) were detected using ultra-performance liquid chromatography (UPLC, tuning) coupled to a mass spectrum (fast atom bombardment mass spectrometry (FAB-MS)); the molecular structure (C_29_H_48_O) of compound **4** corroborated the molecular ion of *m*/*z* 412 [M]+ and 414 [M]+ for compound **5** (Figure 1, Table 1). Stigmasterol (**4**) and β-sitosterol (**5**) were identified by a comparison of the ^1^H and ^13^C nuclear magnetic resonance (NMR) spectra (Table S1) with those reported in the literature [25]. Stigmasterol was identified in the dichloromethane extract from the aerial tissues of the *S. angustifolia* wild plant, and it was reported to have anti-inflammatory and immunomodulatory activities [6].

From the dichloromethane–methanol extract of the SaTRN7.1 hairy root (line 2), the compounds stigmasterol (**4**) and β-sitosterol (**5**) were detected (Table 1) using UPLC, tuning coupled to a mass spectrum (FAB-MS *m*/*z* 412 [M]+ and 414 [M]+) in the sub-fraction SaL2F2R4 (4 mg). It was confirmed that one compound was stigmasterol C_29_H_48_O from its molecular weight of 412 g/mol, and another one was β-sitosterol C_29_H_50_O from its molecular weight of 414 g/mol.

#### 2.2.2. Isolation and Identification of Iso-Sphaeralcic Acid (**6**) and 8-Methyl-Iso-Sphaeralcic Acid (**7**)

**Figure 2:**
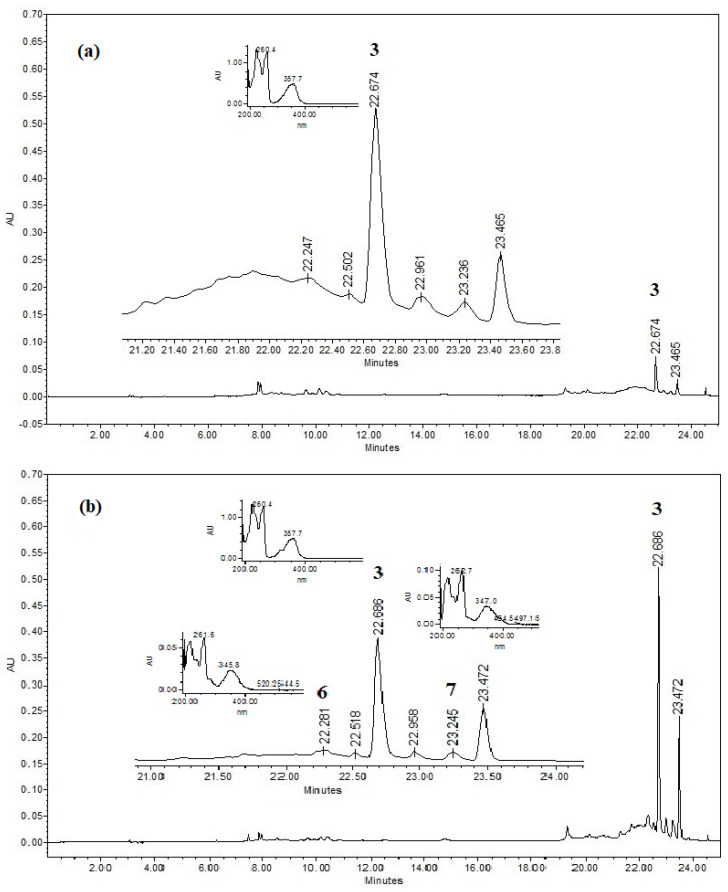
Chromatograms of dichloromethane–methanol extracts from the (**a**) SaTRN12.2 (line 1, 3 mg/mL) and (**b**) SaTRN7.1 (line 2, 0.3 mg/mL) hairy root lines of *S. angustifolia* at λ = 357 nm. It should be noted that in line 1, the compounds were not detected at a concentration of 0.3 mg/mL.

The high-performance liquid chromatography (HPLC) profiles of the dichloromethane–methanol extracts of the hairy root lines of *S. angustifolia* (Figure 2), 1 (3 mg/mL), and 2 (0.3 mg/mL), showed a similarity in chemical constituents of a low polarity, with retention times of 22.28, 22.51, 22.68, 22.95, 23.24, and 23.47 min. It should be noted that in line 1, these compounds were not detected at a lower extract concentration (0.3 mg/mL). The sphaeralcic acid (**3**) standard corresponded to that at 22.68 min. The UV absorption data for the compounds at 22.28 min and 23.24 min (Figure 2) were identical to those shown for compound **3** (22.68 min, λmax = 222, 260 and 357 nm); both compounds **6** (22.28 min) and **7** (23.24 min) were isolated and identified from SaTRN7.1 hairy root (line 2).

Compound **6** was purified as a green powder soluble in methanol from the sub-fraction SaL2F6-8R9 (5 mg). The UV spectrum of compound **6** revealed an absorption spectrum (λ_max_ = 222, 261 and 345 nm) similar to compound **3**. In the positive ion FAB-MS of **6**, quasimolecular ion peaks at *m*/*z* 329 [M + K]^+^ and 272 [M–H_2_O]^+^ were observed, and the HRFAB-MS analysis revealed the molecular formula to be C_16_H_16_O_4_ (*m*/*z* = 272.1219 [M-H_2_O]^+^). Through the analysis of the spectra of one and two dimensions (Table 2) and the comparison with the data described in [18], this compound corresponded to 2-(4,8-dihydroxy,3-isopropyl, 6-methyl, 7-methoxy) naphthoic acid (**6**), called iso-sphaeralcic acid (Figure 1).

**Figure 3:**
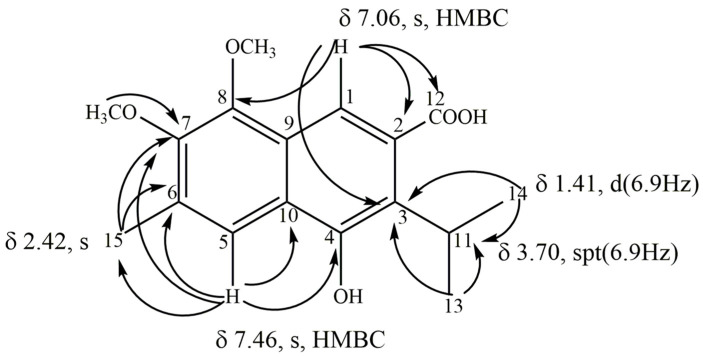
HMBC correlations of compound **7,** named 8-methyl-iso-sphaeralcic acid.

Compound **7** was obtained from the sub-fraction SaL2F10-12R18 as a yellow solid soluble in methanol. In the positive-ion FAB-MS of **7**, quasimolecular ion peaks at *m*/*z* 286[M-H_2_O]^+^ and 271[M–H_2_O-CH_3_]^+^ were observed, and the HRFAB-MS analysis revealed the molecular formula to be C_17_H_18_O_4_ (*m*/*z* = 286.1454 [M-H_2_O]^+^). The UV absorption spectrum (λmax = 214, 262, and 347 nm) was similar to that of compounds **3** and **6**; therefore, it has been proposed to be a derivative of sphaeralcic acid [18]. The ^1^H NMR spectrum showed two singlet signals for aromatic rings at δ 7.06 and δ 7.46, which were assigned according to the heteronuclear single quantum coherence (HSQC) spectrum, and the carbon signals at δ 110.9 and 118.7 were assigned to H-1 and H-5, respectively, for a skeleton naphthalene type. The H-1 (δ 7.06) in the heteronuclear multiple bond correlation (HMBC) experiments were correlated with the carbon signals at δ 29.6, 106.9, 119.5, 158.4, and 167.9, assigned to C-11, C-2, C-3, C-8, and C-12, respectively. The signal H-5 (δ 7.46) in the HMBC was associated with the carbon signals at δ 156.0, 142.8, 130.8, 130.3, and 17.7, and assigned to C-4, C-7, C-6, C-10, and C-15 (Figure 3), respectively. On the other hand, a singlet signal of a methyl group attached to an aromatic ring was observed at δ 2.42 (H-15) and was correlated in HSQC with the carbon signal at δ 17.9, assigned to C-15. The H-15 proton (δ 2.42) in the HMBC was correlated with carbon signals at δ 118.7, 130.2, and 141.2, which were assigned to C-5, C-6, and C-7, respectively. In addition, two singlet oxygen base signals were observed at δ 4.09 and 4.23, corresponding to a methoxyl group (δ 57.8, OCH_3_-7 and 59.9, OCH_3_-8), and an isopropyl unit at δ 1.41 (2CH_3,_ d, 6.9 Hz, H-13 and H-14) and 3.7 (CH, spt, 6.9 Hz, H-11). Finally, ^13^C NMR showed the presence of a carboxylic acid at δ 167.9 (C-11), which was correlated in HMBC with H-1 (δ 6.96) (Figure 3).

Based on an analysis of the spectroscopic data of single and two-dimensional NMR spectroscopy (Table 2) and a comparison with the data described in the literature [18], this compound was determined to be 2-(4-hydroxy,3-isopropyl,6-methyl, 7,8-dimethoxy) naphthoic acid (Figure 1), named 8-methyl-iso-sphaeralcic acid (**7**). In addition, the ^1^H and ^13^C NMR spectra (Table 2) showed the same signals as those described for compound **6**, except for the presence of a methoxyl group. This work is the first report of compounds **6** and **7** isolated from the hairy root cultures of *S. angustifolia*.

The development of a methodology to generate hairy root cultures represents a technological approach to produce metabolites of pharmacological importance, considering that the transformed roots have a higher growth rate and accumulate high levels of active compounds compared with the wild plants [26]. Oncogenes are essential modulators of plant growth and cell differentiation, and their ability to improve secondary metabolite production in transformed cells has been described [27]. Four T-DNA *role* oncogenes (ORFs), called *rolA*, *rolB*, *rolC*, and *rolD*, are critical for the induction, growth, and morphology of hairy roots in infected plants. The hairy root transformation of *S. angustifolia* (SaTRN12.2, line 1, and SaTRN7.1, line 2) was previously confirmed by the presence of the *rolC* gene [24]. The *rolC* gene has an important role as a modulator of secondary metabolite production among medicinal plants [27].

Hairy root culture has many advantages for producing high-value metabolites because of their fast growth and biosynthetic stability for many successive generations [28,29,30]. In addition, this culture could produce new compounds not produced by the plant, for example, from *Lopezia racemose,* the new compound identified was (23R)-2α,3β,23,28-tetrahydroxy-14,15-dehydrocampesterol [31] or cadaverin, an amine detected for the first time in the hairy roots of *Brugmansia candida* [32]. The hairy root culture of *S. angustifolia* produced two new compounds (**6** and **7**) derived from naphthoic acid and sphaeralcic acid [18], not yet identified in the wild plant or in the cells in suspension.

A relationship was reported between the base chemical structure of the plant compound groups and the pharmacological activity. It has been suggested that iso-sphaeralcic acid (**6**) and 8-methyl-iso-sphaeralcic acid (**7**) could have anti-inflammatory and immunomodulatory effects similar to the activities shown for sphaeralcic acid in acute and chronic inflammation models in mice [20,23].

The galphimines A and B (nor-seco-triterpenes) isolated from *Ghalphimia glauca*, and their synthetic derivatives, have presented anxiolytic effects in mice, considering that the determining factor of this activity was attributed to the presence of hydroxyl groups in C-4, C-6, and C-7 and the presence of a double bonding in the ring A [33]. In the same way, the anti-inflammatory effect of galphimine-A and galphimine-E from *G. glauca* is related to the presence of an oxygenated function group in C-6 [34].

Reports of the structure–activity relationship of flavonoids as antibacterial agents have suggested that hydroxyl groups at special sites on aromatic rings improve the activity. Instead, methylation of the active hydroxyl groups decreases the activity. The phenyl groups, alkyl-amino chains, alkyl chains, and nitrogen or oxygen also enhance the activity of flavonoids [35]. Similarly, the relationship between the structural characteristics of flavonoids shows that the substitution pattern of free hydroxyl groups on the flavonoid skeleton helps determine the free radical scavenger potential [36].

### 2.3. Gastroprotector Effect of Dichloromethane–Methanol Extracts from SaTRN7.1 Hairy Roots Line of S. angustifolia

In this study, the effects of dichloromethane–methanol extract from the SaTRN7.1 hairy root (line 2) of *S. angustifolia* on ethanol-induced gastric ulcers in mice was examined. The mice administered with the vehicle (1% Tween-20) presented stomachs with an average ulcerated index of 0.664 (Table 3). The mice treated with omeprazole and dichloromethane–methanol extract (100 mg/kg) of the hairy root presented stomach areas with less damage and smaller ulcer indices according to the ANOVA and Dunnette’s test. According to Student’s T test (*p* < 0.05), the extract showed a protective activity against gastric ulcers induced with ethanol, superior to the effect of omeprazole (1 mg/kg). The oral administration (100 mg/kg) of the dichloromethane–methanol extract inhibited 92% ulcer development. The extract contained mainly sphaeralcic acid, a compound with anti-inflammatory and immunomodulatory properties, sphaeralcic acid isomers (iso-sphaeralcic acid, **6** and 8-methyl-iso-sphaeralcic acid, **7**), and stigmasterol and β-sitosterol. Stigmasterol is a vegetal sterol with anti-osteoarthritic, anti-hypercholesterolemia, cytotoxic, anti-tumoral, hypoglycemic, anti-mutagenic, antioxidant, and anti-inflammatory effects [37]. Stigmasterol acts as an anti-inflammatory that reduces the pro-inflammatory production of pro-inflammatory molecules, which contributes to osteoarthritis-induced cartilage degradation [38,39]. In addition, it had an analgesic effect in synergy with 9-hexacosane [40]. β-sitosterol was reported to have immunomodulatory [41] and anti-inflammatory properties on mouse models of edema auricular induced with TPA [42] and paw edema induced with λ-carrageenan [43]. The hairy root culture of *S. angustifolia* is proposed as a relevant source of new molecules with possible anti-inflammatory and gastroprotective activities. It will be important to evaluate the iso-sphaeralcic acid (**6**) and 8-methyl-iso-sphaeralcic acid (**7**) in acute and chronic inflammation models in mice, as well as sphaeralcic acid and its isomers in ethanol-induced gastric ulcers in mice.

## 3. Materials and Methods

### 3.1. S. angustifolia Hairy Root Cultures

The SaTRN12.2 (1) and SaTRN7.1 (2) hairy root lines of *S. angustifolia* were previously established by infecting internodes with *Agrobaterium rhizogenes* [24] and were provided by the research group of Dr. Nicasio-Torres.

The hairy roots (10 g) were grown in 1 L Erlenmeyer flasks with 400 mL of phyto-regulator-free MS culture medium [24,44] and 3% sucrose. The cultures were incubated at 26 °C with a photoperiod of 16 h light under 50 μm/m^2^/s of fluorescent white light intensity and 8 h of darkness, and orbital agitation at 110 rpm (New Brunswick Scientific Co., Edison, NJ, USA). The hairy roots were transferred to fresh medium every three weeks using a 6% inoculum. At this time, each hairy root line was vacuum filtered separately using a Buchner funnel (Whatman filter paper No. 1, 9-cm diameter), and the retaining roots were weighed and transferred to flasks with fresh medium, preserving the same inoculum for its growth and proliferation.

### 3.2. Chemical Profiles of Dichloromethane–Methanol Extracts from S. angustifolia Hairy Roots Lines

#### 3.2.1. Extract Preparation of Hairy Root Lines

Flasks of each hairy root line were vacuum filtered using a Buchner funnel (Whatman filter paper No. 1, 9-cm diameter). First, the retained roots were washed with sterile distilled water, and the harvested roots were then dried in an oven (Thelco 160 DM) at 65 °C for 48 h. Next, the dry and ground hairy root lines (173 g SaTRN12.2 and 100 g SaTRN7.1) were extracted thrice by maceration (24 h for each procedure) at room temperature, with a mixture of reactive grade solvents (dichloromethane–methanol 9:1; Merck) at a ratio of 1:50 (*w*/*v*). Finally, the extracts were filtered, pooled, and concentrated to dryness under reduced pressure in a rotary evaporator [24].

#### 3.2.2. Quantification of Scopoletin and Sphaeralcic Acid

The dichloromethane–methanol extracts of both hairy root lines, SaTRN12.2 (1) and SaTRN7.1 (2), were analyzed using HPLC by monitoring scopoletin (**1**) absorbance at λ = 344 nm and sphaeralcic acid (**3**) at λ = 357 nm. Scopoletin (99% purity; Sigma–Aldrich Chemical) and sphaeralcic acid (95% purity) were identified by comparing their absorption spectra and their retention times (scopoletin, 10.3 min, and sphaeralcic acid, 22.8 min). Sphaeralcic acid was purified from cells in a suspension of *S. angustifolia* and was identified by our group [18,19,20]. The concentration ratio for scopoletin quantification ranged from 1.25 to 20 µg/mL, and for sphaeralcic acid from 2.5 to 40 µg/mL. The regression equation for scopoletin was (y) = 165,407 (x) + 16,720, r^2^ = 0.9993, and for sphaeralcic acid it was (y) = 7381.9 (x) + 1362.2, r^2^ = 0.9998.

#### 3.2.3. HPLC Conditions

The HPLC analyses were performed using a Waters system (2695 Separation Module) coupled to a diode array detector (2996) with a 190–600-nm detection range and were operated through the Manager Millennium software system (Empower 1; Waters Corp.). The separations were performed on a Spherisorb RP-18 column (250 × 4.6 mm, 5.0 µm; Waters Corporation) using a constant temperature of 25 °C during the analysis. Samples (20 µL) were eluted at a flow rate of 1.0 mL/min with a mobile phase gradient of high purity: (A) water with trifluoroacetic acid (0.5%, Sigma–Aldrich, St. Louis, MO, USA) and (B) acetonitrile (Merck, Darmstadt, Germany). The mobile phase was started with water (100%) and was maintained for 1 min. The concentration of solvent B was increased gradually to 5% (at 2 min), 30% (at 12 min), 50% (at 4 min), and 80% (at 1 min). During the next 2 min, solvent B was increased to 100%. Finally, the next 3 min were used to return the mobile phase to the initial condition. The chromatographic method had a 25 min run time [18,19,20,21,22,23,24].

#### 3.2.4. Isolation of Compounds from the SaTRN12.2 (Line 1) and SaTRN7.1 (Line 2) Hairy Roots

The extract from line 1 (3 g) was fractionated by chromatography on an open glass column (2.5 × 44 cm) packed with silica gel (25 g, Merck) and a dichloromethane–methanol gradient elution system with 5% polarity increments of methanol and the collection of 10 mL aliquots, yielding 30 fractions. The analysis by TLC allowed for the pooling of aliquots in 10 fractions (SaL1F1 to SaL1F10). Fraction SaL1F3 (0.089 g) was purified by column chromatography (1.5 × 5 cm) on silica gel (1 g, RP-18, Merck) using a water–acetonitrile gradient system with polarity changes of 10% and volumes of 10 mL, to give 40 fractions. Analysis by TLC allowed for pooling into seven sub-fractions (SaL1F3R1 to SaL1F3R7). In the SaL1F3R6 sub-fraction (12 mg), a single spot was observed by TLC, indicating a mixture of two compounds (**4** and **5**) identified by ^1^H and ^13^C NMR as stigmasterol and β-sitosterol, respectively.

The extract from line 2 (3.6 g) was fractionated by glass open column chromatography (2.5 × 44 cm) packed with silica gel (25 g, 70–230 mesh, Merck) and an *n*-hexane–ethyl acetate gradient elution system with polarity increments of 10%. Aliquots of 10 mL were collected (78), and 16 fractions (SaL2F1 to SaL2F16) were pooled according to their chemical profile observed by TLC.

The SaL2F2 fraction (0.03 g) was purified by column chromatography (1.5 × 21 cm) of silica gel (5 g) using a dichloromethane–acetone system with polarity changes of 5%, to provide 22 sub-fractions of 10 mL. After the TLC analysis, the samples were grouped into eight sub-fractions (SaL2F2R1 to SaL2F2R8). In sub-fraction SaL2F2R4, a mixture of stigmasterol (**4**) and β-sitosterol (**5**) was identified.

Fractions SaL2F6-SaL2F8 (0.243 g) were pooled and purified by column chromatography (1.5 × 21 cm) of silica gel (8 g) with *n*-hexane–ethyl acetate and polarity changes of 1% to provide 65 sub-fractions of 10 mL. They were pooled in 13 sub-fractions (SaL2F6-8R1 to SaL2F6-8R13). In the subfraction SaL2F6-8R2, sphaeralcic acid (**3**) was isolated and identified, and in the SaL2F6-8R9 subfraction a green powder was obtained that was identified as compound **6** (iso-sphaeralcic acid).

Fractions SaL2F10-12 (0.036 g) were pooled and purified by column chromatography (1.5 × 34 cm) of silica gel RP-18 with a water–acetonitrile system with changes of 10% to give 90 samples of 10 mL. According to the TLC analysis, they were pooled in 25 sub-fractions (SaL2F10-12R1 to SaL2F10-12R25). In fraction SaL2F10-12R18, a yellow solid was obtained, which was identified as compound **7** (8-methyl-iso-sphaeralcic acid).

#### 3.2.5. NMR Equipment and Masses

All NMR spectra were recorded on Agilent DD2-600 at 600 MHz, for ^1^H NMR, ^1^H-^1^H COSY, HMQC, and HMBC, and 150 MHz for ^13^C NMR and ^13^C DEPT, using CDCl_3_ and CD_3_OD as solvents. Chemical shifts were reported in ppm relative to TMS. FAB-MS and HRFAB-MS were performed using a JEOL MS-700 mass spectrometer.

### 3.3. Gastric Ulcers Induced with Absolute Ethanol in Mice

Female ICR mice (35–40 g, Envigo) were integrated with 10 animals per cage and were preserved in the bioterium at 25 °C, with 12 h light/12 h dark cycles. Water and food (pellets from Harlan Rodent Lab Diet) were provided ad libitum. The ethical use of animals was conducted following with the Mexican Official Regulation dating from 1999 (NOM-062-ZOO1999). The research protocol was approved by the Local Committee for Research in Health (CLIS-1702) and Ethical Committee (CONBIOETICA 17 CEI 00120190121) of Instituto Mexicano del Seguro Social (IMSS) on 13 July 2021, with registration number 2021-1702-06.

Mice weighing between 35 and 40 g were divided into groups of six mice each. The mice were deprived of food with free access to drinking water 24 h prior to experimentation. After fasting, the mice were pre-treated with a single dose of vehicle (1% Tween-20; 0.1 mL/10g), omeprazole (20 mg/kg), and dichloromethane–methanol extract (100 mg/kg) of the SaTRN7.1 hairy root (line 2). Gastric lesions were induced with absolute ethanol (0.10 mL/10g body weight) 1 h after administering the treatments [45]. The mice were anesthetized with Sedalphorte (pentobarbital sodium 25 mg/kg) administered intraperitoneally after 1 h of ulcer induction, and then sacrificed by cervical dislocation. The stomachs were removed, opened along the greater curvature, and rinsed gently with water to remove the gastric contents. Ethanol-induced ulcers appeared as elongated bands of hemorrhagic lesions in the glandular region. After identifying the ulcers (damaged area), photographs were taken of each stomach, and the damaged area was measured from the photographs by planimetry using ImageJ software. The area of each ulcer lesion was measured by counting the number of small squares, 0.1–1 mm, covering the length and width of each ulcer. In each group, the area sum of all lesions for each stomach was used for the ulcerated area (UA) calculation (mm^2^). The ulcer index (UI) was estimated by dividing the UA by the stomach total area (mm^2^). The level of protection (%) was determined by the UI of the control minus the UI of the treated group divided by the UI of the control per 100.

$$\mathrm{UI}=\left(\frac{\mathrm{UA}}{\mathrm{stomach}\;\mathrm{total}\;\mathrm{area}}\right)\;\%\;\mathrm{Protection}=\left[\frac{\mathrm{UI}\;\mathrm{control}-\mathrm{UI}\;\mathrm{treated}\;\mathrm{group}}{\mathrm{UI}\;\mathrm{control}}\right]\times 100$$

## 4. Conclusions

The hairy root lines (SaTRN12.2 and SaTRN7.1) of the *S. angustifolia* plant are producers of the anti-inflammatory and immunomodulatory compounds scopoletin, sphaeralcic acid, stigmasterol, and β-sitosterol, and two isomers of sphaeralcic acid have been described for the first time: iso-sphaeralcic and 8-methyl-iso sphaeralcic. Furthermore, the protective activity against gastric ulcers induced with ethanol of the dichloromethane-methanol extract of SaTRN7.1 confirmed the traditional use of the *S. angustifolia* plant to treat gastrointestinal illness; this extract, similar to the plant, contains mainly compounds with an anti-inflammatory activity and gastroprotective effect similar to scopoletin and β-sitosterol. Biotechnologically, stable and enhanced active compound production of the SaTRN7.1 hairy root culture could be scaled up using a bioreactor.

## Figures and Tables

**Table 1:** Compounds identified and isolated from the dichloromethane–methanol extracts of the SaTRN12.2 (line 1) and SaTRN7.1 (line 2) hairy root lines of *Sphaeralcea angustifolia*.

Hairy Root Lines			
SaTRN12.2 (Line 1)		SaTRN7.1 (Line 2)	
Scopoletin	0.0022 mg g^−1^		
Sphaeralcic acid	0.22 mg g^−1^	Sphaeralcic acid	3.07 mg g^−1^
Stigmasterol		Stigmasterol	
β-sitosterol		β-sitosterol	
		Iso-sphaeralcic acid	
		8-methyl-iso-sphaeralcic acid	

**Table 2:** ^1^H and ^13^C NMR spectroscopic data (600 MHz, δ ppm) for compounds **6** and **7**.

Position	δ ^1^H (*J* in Hz, CD_3_OD) (6)	δ ^13^C, (CD_3_OD) (6)	δ ^1^H (*J* in Hz, CDCl_3_) (7)	δ ^13^C, (CDCl_3_) (7)
1	6.96, s	115.7	7.06, s	110.9
2		106.1		106.9
3		120.2		119.5
4		156.0		154.8
5	7.49, s	119.7	7.46, s	118.7
6		130.8		130.2
7		142.8		141.2
8		158.1		158.4
9		123.8		121.5
10		130.3		129.6
11	3.67, spt (6.9)	30.3	3.7, spt (6.9)	29.6
12		170.4		167.9
13	1.37, d (6.9)	24.0	1.41, d (6.9)	23.8
14	1.37, d (6.9)	24.0	1.41, d (6.9)	23.8
15	2.40, s	17.7	2.42, s	17.9
OCH_3_-7	4.0, s	60.7	4.09, s	57.8
OCH_3_-8			4.23, s	59.9

**Table 3:** Effect gastroprotector of dichloromethane-methanol extract from *Sphaeralcea angustifolia* hairy root against ethanol-induced gastric lesions in mice.

Treatment	Dose (mg/kg)	Ulcer Index	Protection (%)
Vehicle	1% Tween	0.664 ± 0.044	-
Omeprazole	20	0.403 ± 0.067 **	39.30 ± 10.05
SaTRN7.1 hairy root (line 2) CH_2_Cl_2_:CH_3_OH (9:1) extract	100	0.052 ± 0.006 **	92.11 ± 0.93 **

Data presented as means ± SD (n = 6). ** Ulcer index with ** are significantly different according to Dunnette’s test compared to vehicle group (F = 87.79, *p* = 0.0001; Dunnett_0.05_ = 3.04). Protection (%) with ** is highly significantly different between treatments according to Student’s *t* test (*p* < 0.05).

## Data Availability

The data presented in the study are available in the article and in the Supplementary Materials.

## Supplementary Materials

The following supporting information can be downloaded at: https://www.mdpi.com/article/10.3390/plants12051090/s1. Table S1: NMR Spectroscopic data of compounds **4** and **5**. Figures S1 and S2: Isolation of the active compounds from the SaTRN12.2 (line 1) and SaTRN7.1 (line 2) hairy roots of *Sphaeralcea angustifolia*. Figures S3 and S4: Chromatographic elution of compounds **1**, **4**, **and 5**. Figures S5–S7: HPLC chromatogram of compounds **3**, **6**, and **7**. Figures S8–S13: One- and two-dimensional NMR spectra of compound **6**. Figures S14 and S15: Mass spectrum of compound **6**. Figures S16–S20: One- and two-dimensional NMR spectra of compound **7**. Figures S21 and S22: Mass spectrum of compound **7**.
