# Supplementary material for: Production of Two Isomers of Sphaeralcic Acid in Hairy Roots from Sphaeralcea angustifolia
Source: Plants (Basel). 2023 Mar 1;12(5):1090. doi: 10.3390/plants12051090 (PMC10005507; doi:10.3390/plants12051090)
Supplement: Supplementary file 1 [file plants-12-01090-s001.zip › plants-2203823-SI.pdf]

Table S1. NMR Spectroscopic Data (CD_3COCD_3, δ ppm) for stigmasterol y β-sitosterol.

Position	δ [13]C Stigmasterol	δ [1] H Stigmasterol	δ [13]C β-sitosterol	δ [1] H β-sitosterol
1	37.28		37.28	
2	31.7		31.70	
3	71.82	3.52 (1H,m)	71.82	3.52 (1H,m)
4	42.34		42.34	
5	140.78		140.78	
6	121.70	5.35 (1H,m)	121.71	5.35 (1H,m)
7	31.93		31.91	
8	31.93	0.70 (3H,s)	31.88	
9	50.17		50.20	
10	36.53		36.53	
11	21.09		21.07	
12	39.71		39.80	
13	39.8		42.34	
14	56.79		56.79	
15	24.37		24.31	
16	29.20		28.25	
17	55.99		56.09	
18	12.05		11.86	0.68 (3H,s)
19	19.40		19.40	
20	40.47		36.15	
21	21.10		18.79	
22	138.3	5.02 (1H,dd,J=8.6,15.03 Hz)	33.98	
23	129.31	5.15 (1H, dd, J=8.6,15.1 Hz)	26.14	
24	51.25		45.88	
25	31.39		28.90	
26	18.99		19.05	
27	21.21		19.81	
28	25.40		23.10	
29	12.23		12.23	

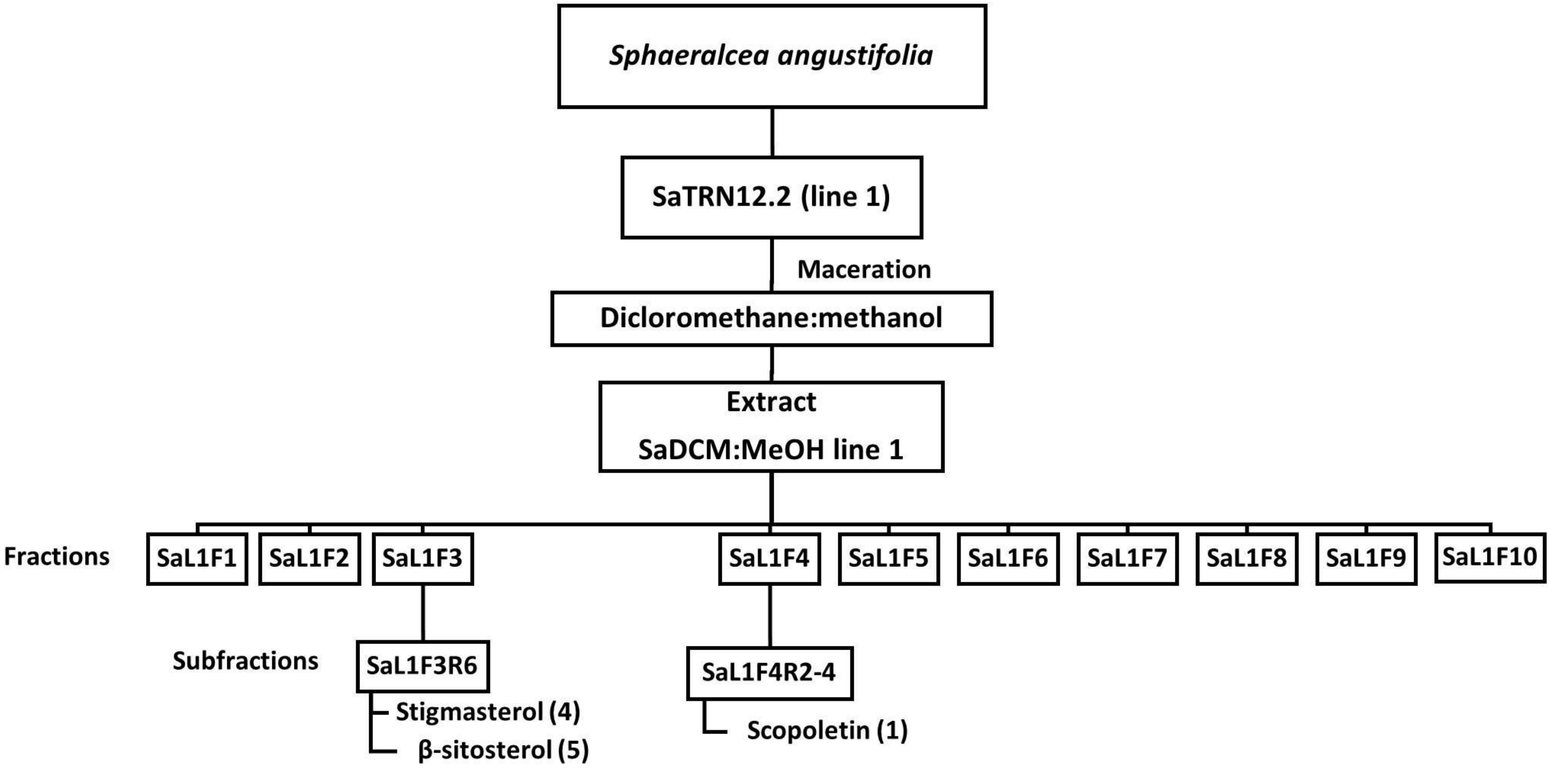

Figure S1. Isolation of the active compounds from the SaTRN12.2 (line 1) hairy roots of *Sphaeralcea angustifolia.*

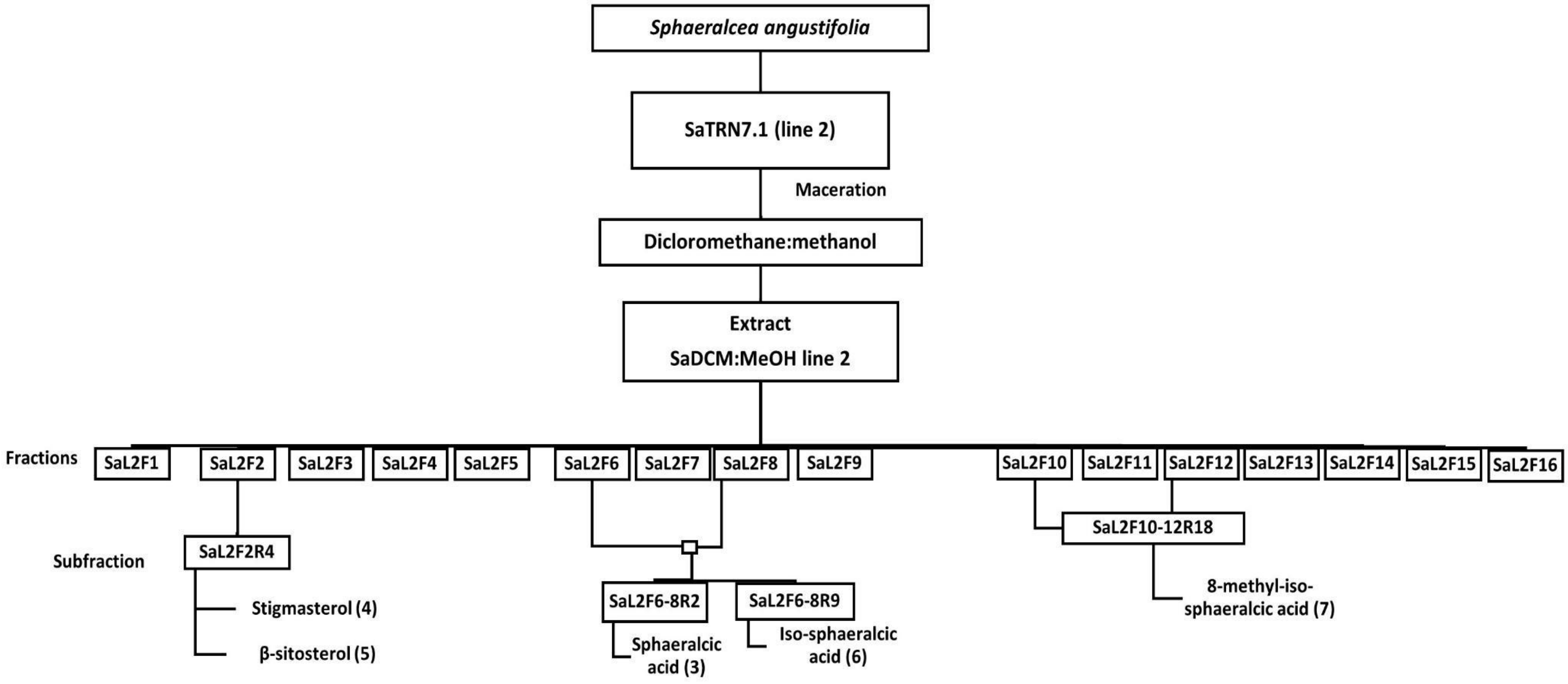

Figure S2. Isolation of the active compounds from the SaTRN 7.1 (line 2) hairy roots of *Sphaeralcea angustifolia.*

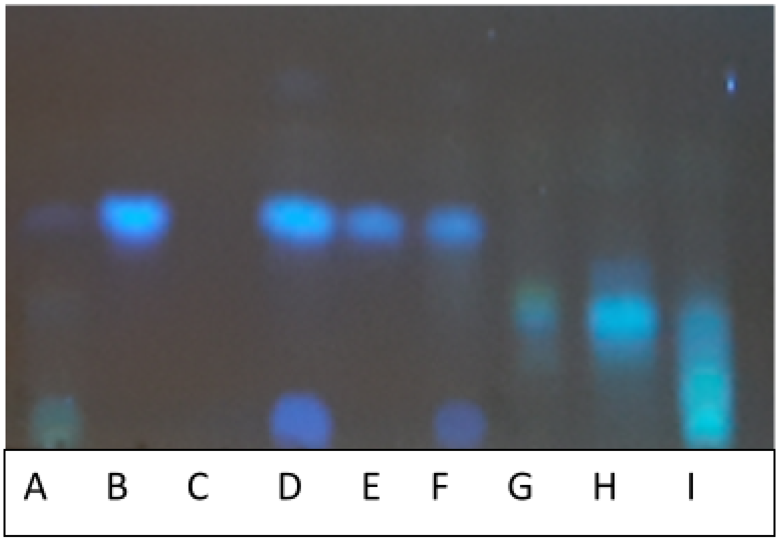

Figure S3. Chromatographic elution of (A) SaL1F4 fraction and (C) 1, (D) 2, (E) 3, (F) 4, (G) 5, (H) 6 and (I) 7 sub-fractions from SaTRN12.2 hairy root dichloromethane-methanol extract (line 1), and B) scopoletin standard under UV λ=365 nm. Elution system 60:40 H_2O:CH_3CN.

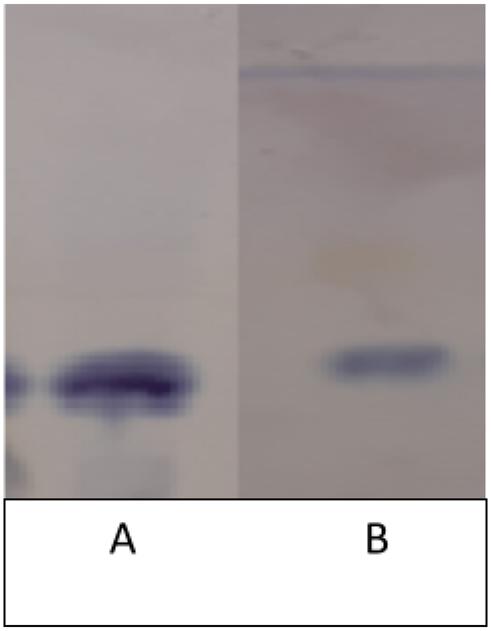

Figure S4. Thin layer chromatography for identification of compounds **4** (stigmasterol) and **5** (β-sitosterol) of (A) SaL1F3R6 sub-fraction of SaTRN12.2 hairy roots (line 1) and (B) SaL2F2R4 sub-fraction of SaTRN7.1 hairy root dichloromethane-methanol extract (line 2) exposed to ceric sulphate. Elution system 80:20 C_6H_{14}:CH_3-COO-CH_2-CH_3.

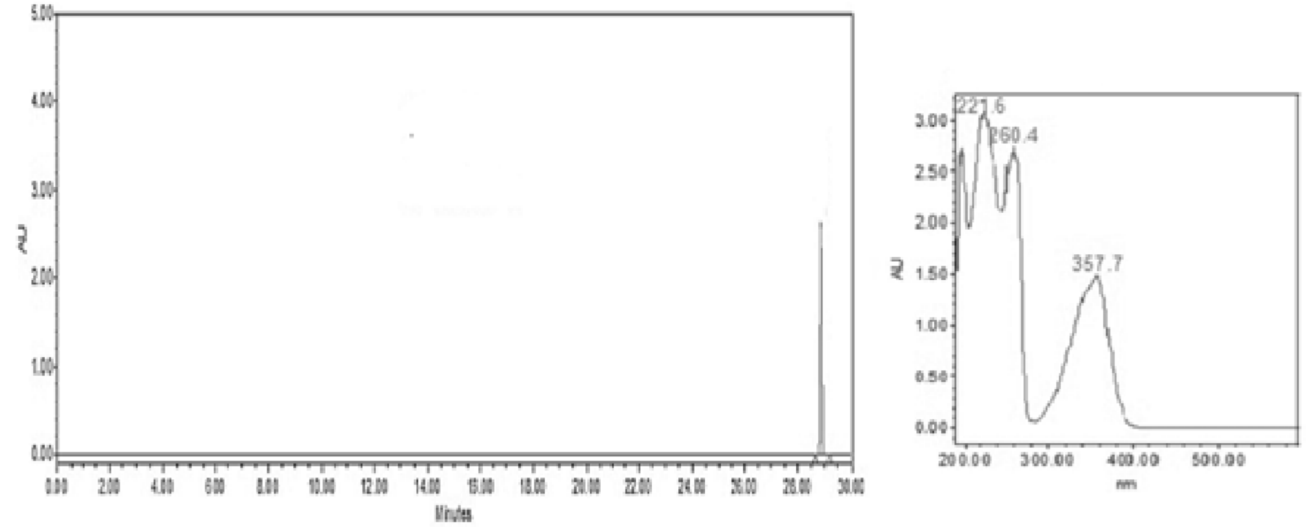

Figure S5. HPLC chromatogram profile at λ =357 nm and absorption spectrum of SaL2F6-8R2 sub-fraction identified as sphaeralcic acid (**3**).

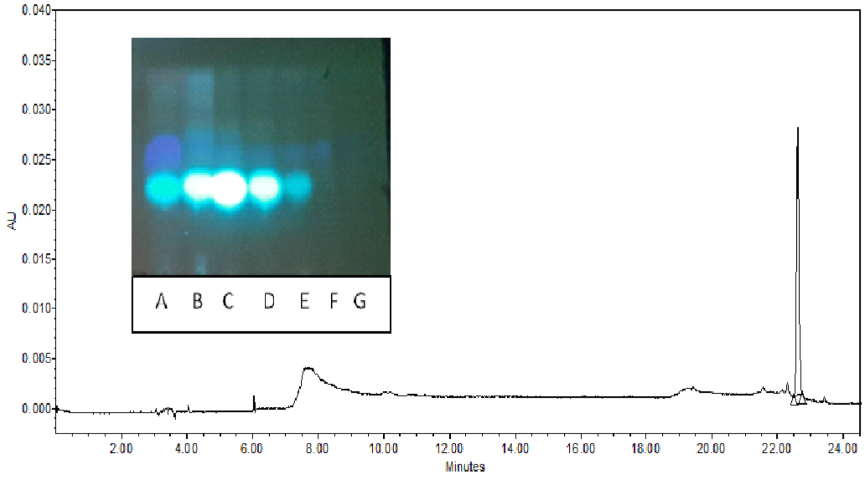

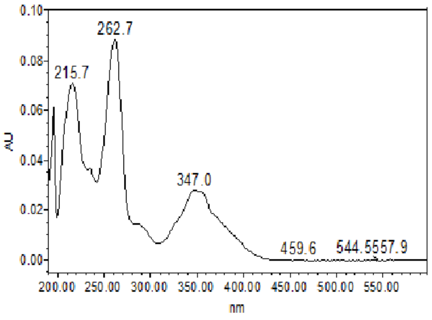

Figure S6. HPLC chromatogram profile at λ =357 nm and absorption spectrum of SaL2F6-8R9 sub-fraction identified as compound **6** (iso-sphaeralcic acid).

Chromatographic elution of (A) SaL2F6-8 fraction and (B) 8, (C)9, (D) 10, (E) 11, (F) 12 and (G) 13 subfractions from SaTRN7.1 hairy roots (line 2) under UV λ=365 nm and an elution system of 80:20 CH_2Cl_2: $CH_3(CO)CH_3$.

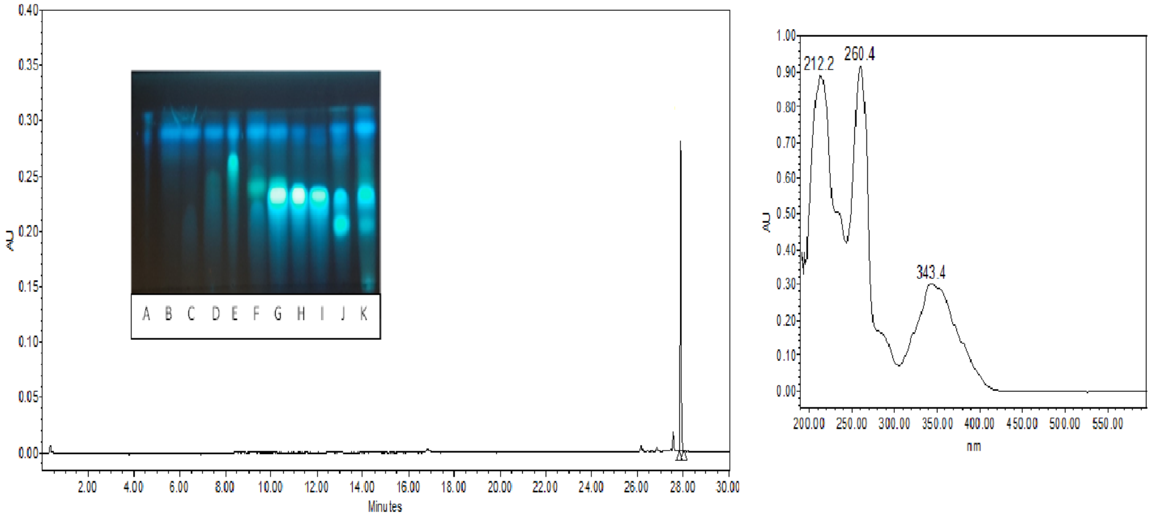

Figure S7. HPLC chromatogram profile at λ =357 nm and absorption spectrum of SaL2F10-12R18 sub-fraction identified as compound **7** (8-methyl-iso-sphaeralcic acid). Chromatographic elution of sub-fractions (A) 10, (B) 11, (C) 12, (D) 13, (E) 14, (F) 15, (G) 16, (H) 17, (I) 18 (J) 19 and (K) SaL2F10-12 fraction from SaTRN7.1 hairy root dichloromethane-methanol extract (line 2) under UV λ=365 nm and an elution system of 30:70 H_2O:CH_3CN.

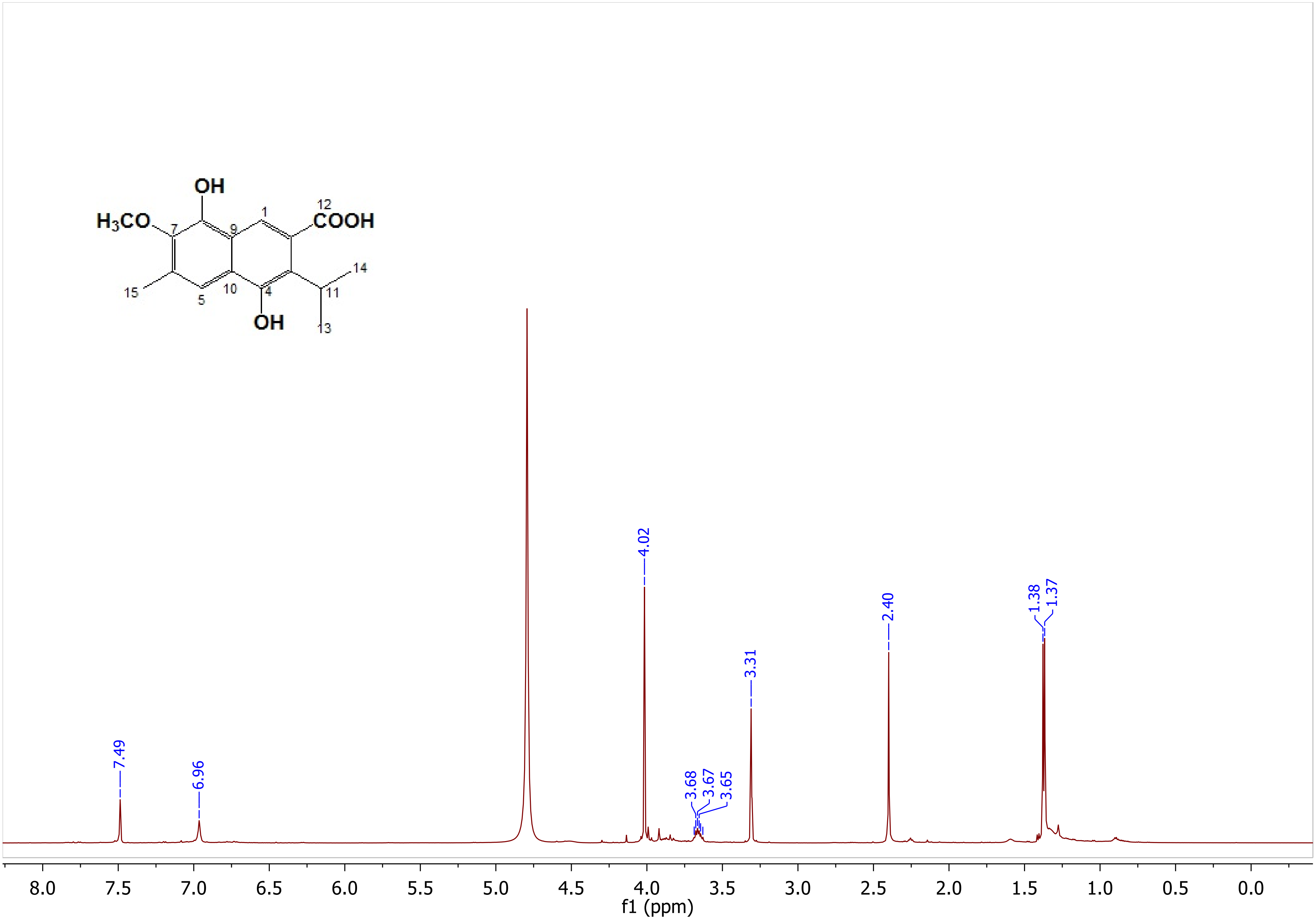

Figure S8. ^{1}H-NMR (CD_3OD, 600 MHz) of iso-sphaeralcic acid (**6**)

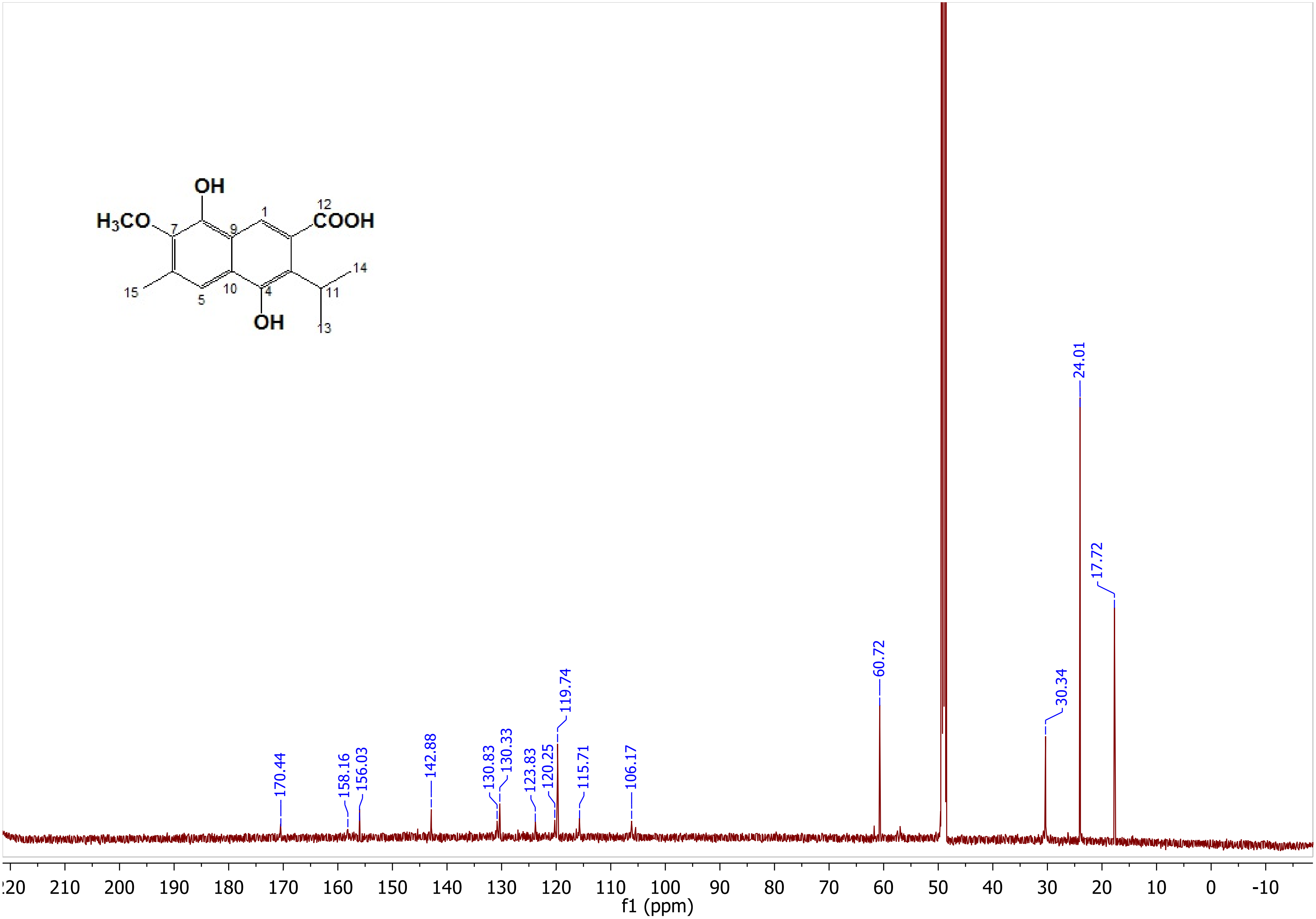

Figure S9. ^{13}C-NMR (CD_3OD, 150 MHz) of iso-sphaeralcic acid (**6**)

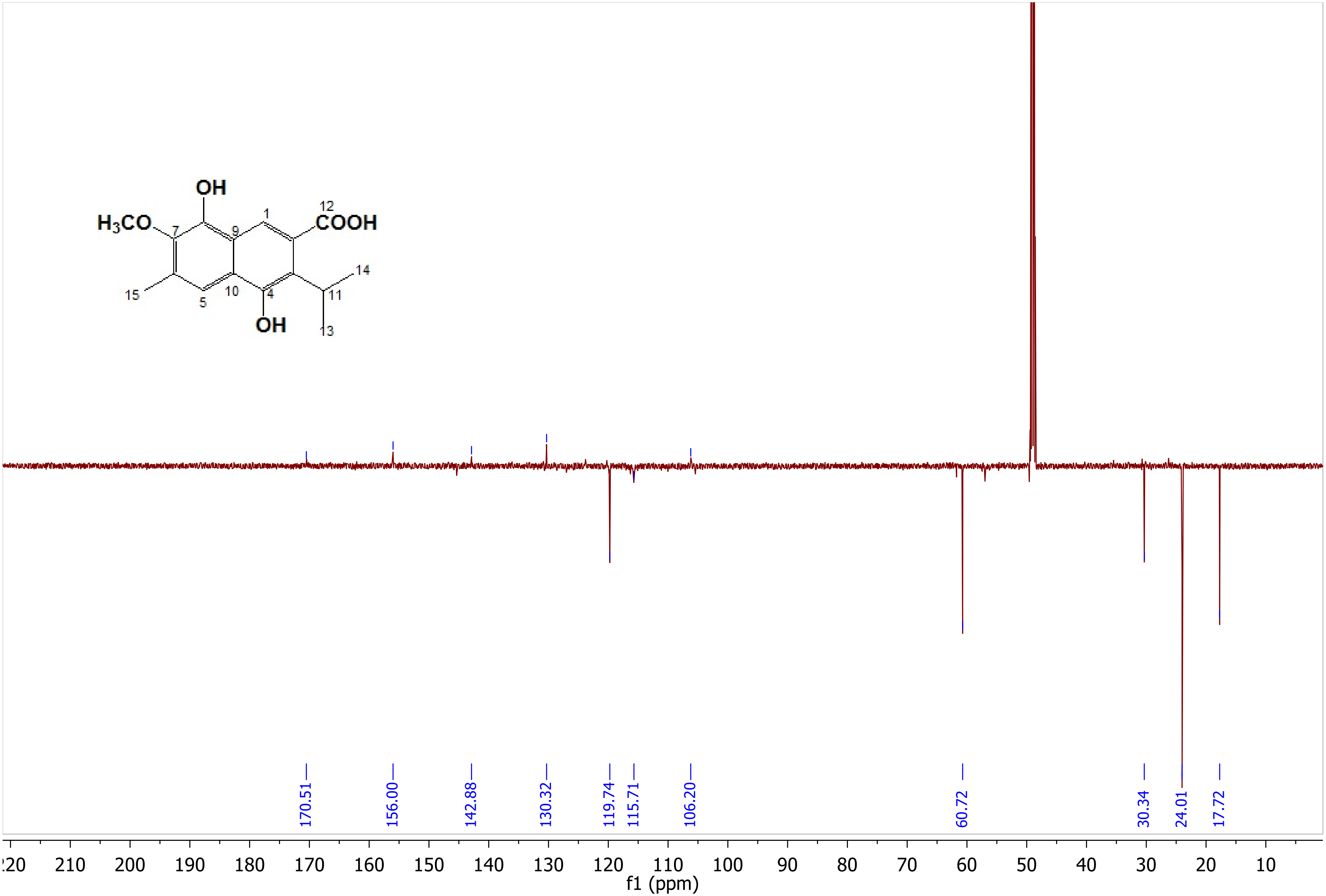

Figure S10. ^{13}C-DEPT NMR (CD_3OD, 150 MHz) of iso-sphaeralcic acid (**6**)

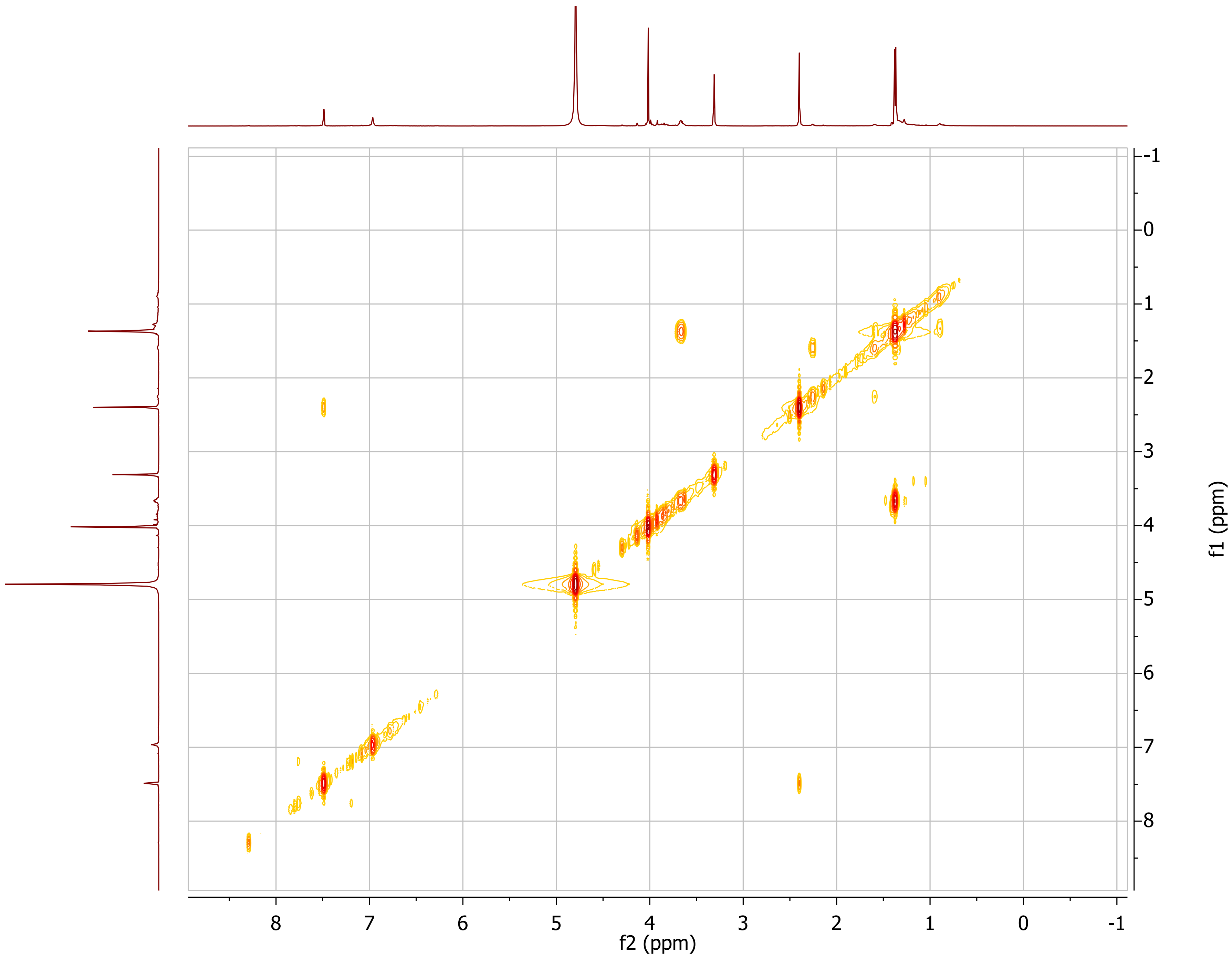

Figure S11. ^{1}H-^{1}H (COSY) NMR (CD_3OD, 600 MHz) of iso-sphaeralcic acid (**6**)

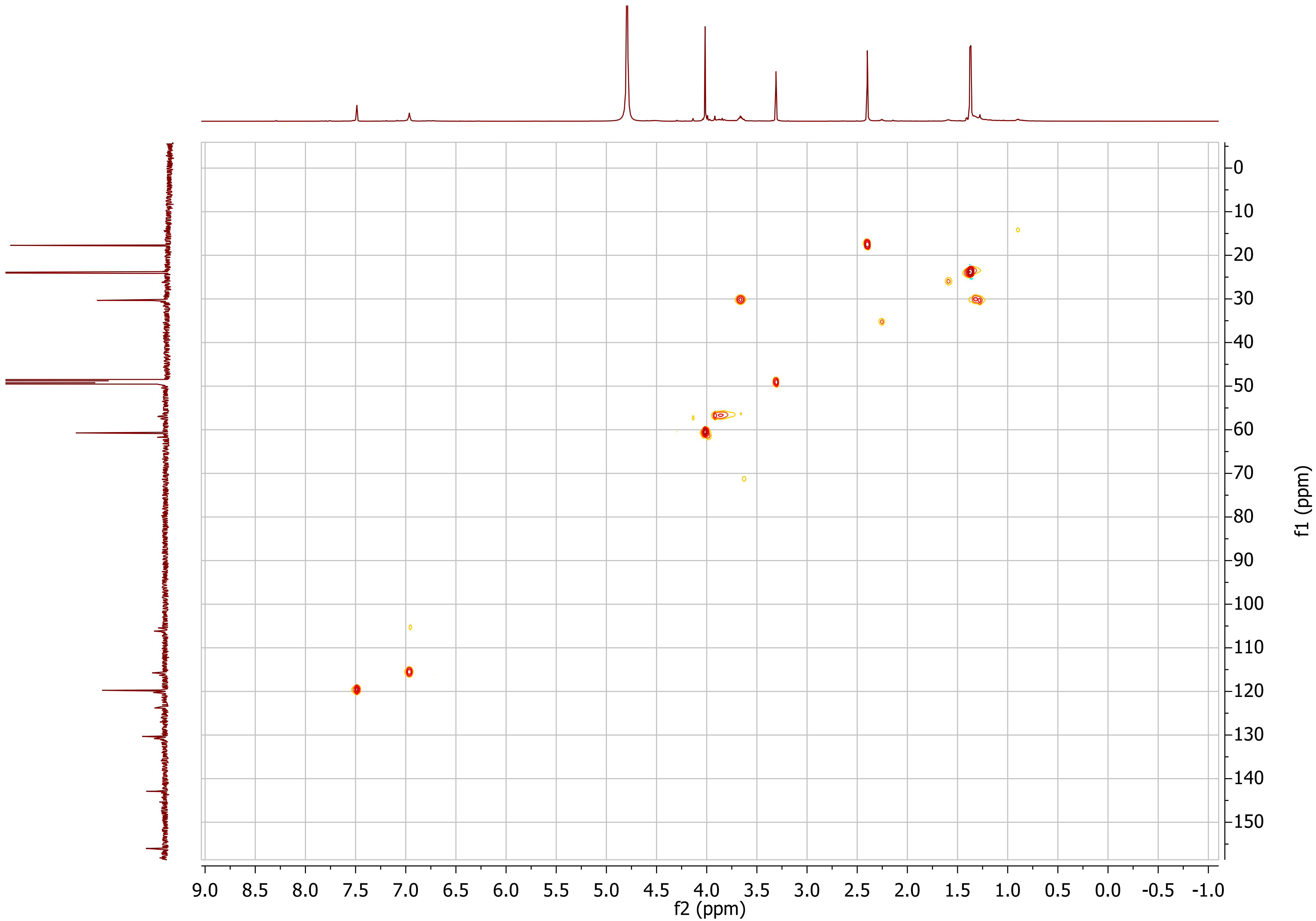

Figure S12. ^{1}H-^{13}C (HSQC) NMR (CD_3OD, 600 MHz) of iso-sphaeralcic acid (**6**)

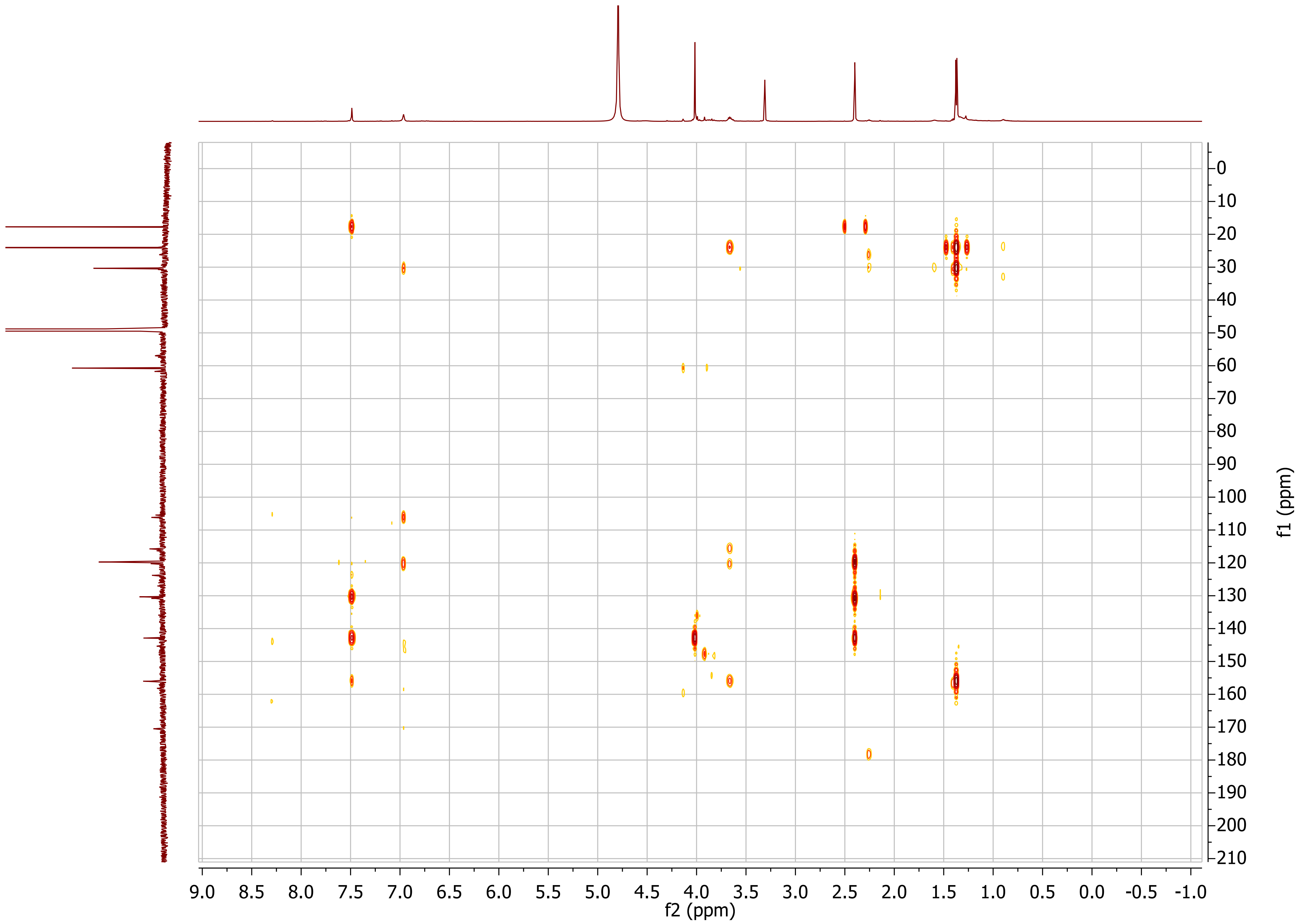

Figure S13. ^{1}H-^{13}C (HMBC) NMR (CD_3OD, 600 MHz) of iso-sphaeralcic acid (**6**)

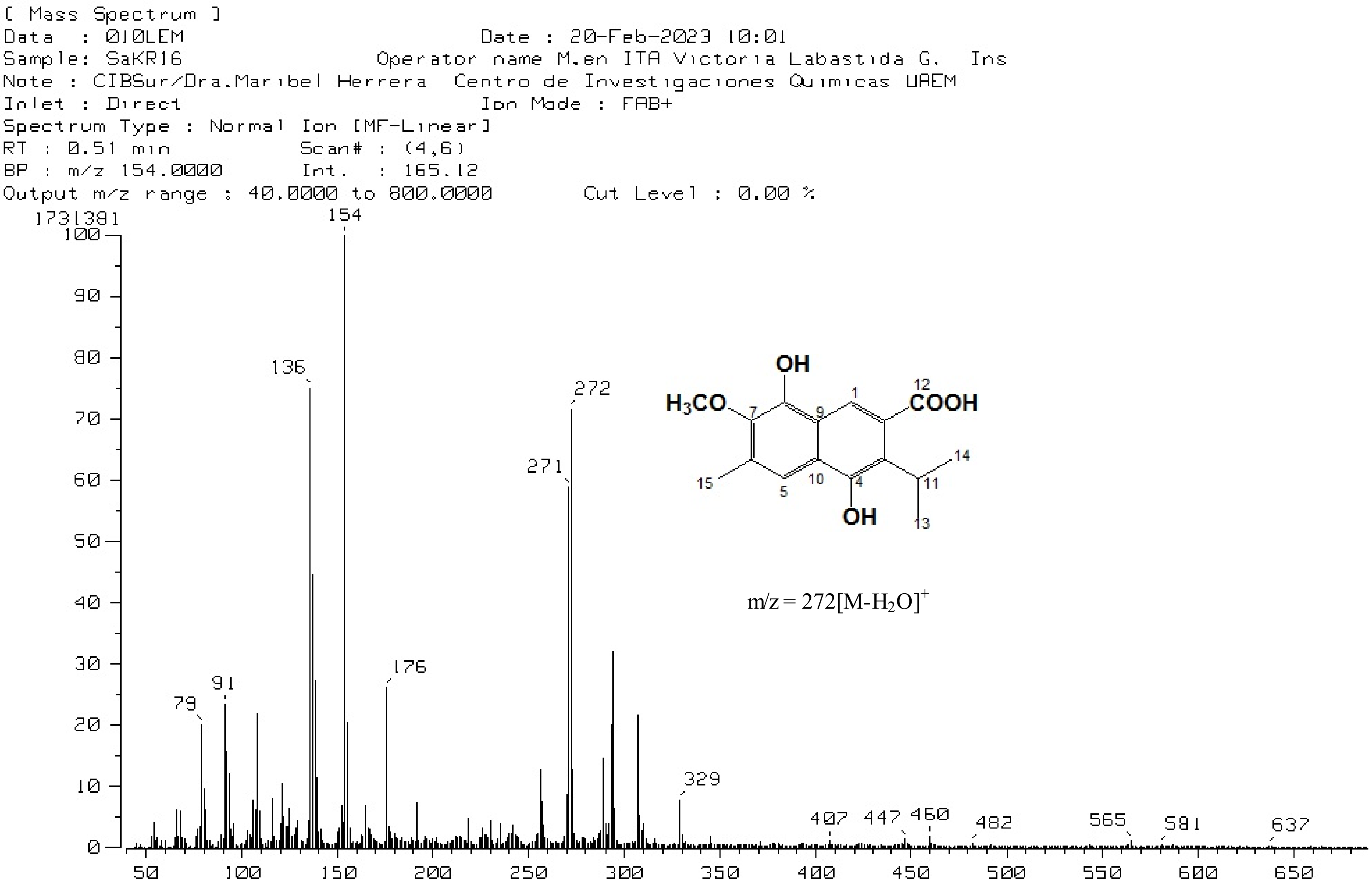

Figure S14. Mass spectrum (FAB-MS) of iso-sphaeralcic acid (**6**)

Servicio de Espectrometría de Masas
MStation JMS-700 JEOL Alta Resolución
Centro de Investigaciones Químicas UAEM
Tel. 329-79-97 ext.6013

UNIVERSIDAD AUTÓNOMA DEL ESTADO DE MORELOS

CENTRO DE INVESTIGACIONES QUÍMICAS

```
[ Elemental Composition ]
Data   : 010LEM-HR                      Date : 20-Feb-2023 10:03

Sample: SaKR16                    Operator name M.en ITA Victoria Labastida G.  I
Note : CIBSur/Dra. Maribel Herrera  Centro de Investigaciones Químicas UAEM

Inlet : Direct                          Ion Mode : FAB+
RT : 0.08 min                           Scan#: 3+(42,49)

Elements : C 40/0, H 49/0, O 5/0
Mass Tolerance      : 1000ppm, 3mmu if m/z < 3, 17mmu if m/z > 17

Unsaturation (U.S.) : -0.5 - 9.0

Observed m/z Int%   Err[ppm / mmu]    U.S.  Composition
  272.1219   100.0    +62.5 / +17.0    9.0  C 16 H 16 O 4
```

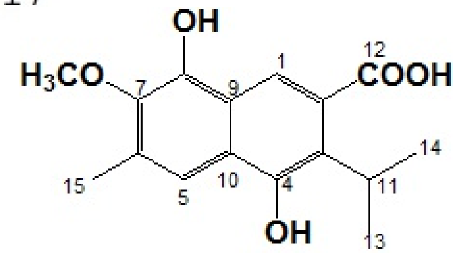

m/z = 272[M-H_2O]$^+$
$C_{16}H_{16}O_4$

Figure S15. High resolution mass spectrum (HRFAB-MS) of iso-sphaeralcic acid (**6**)

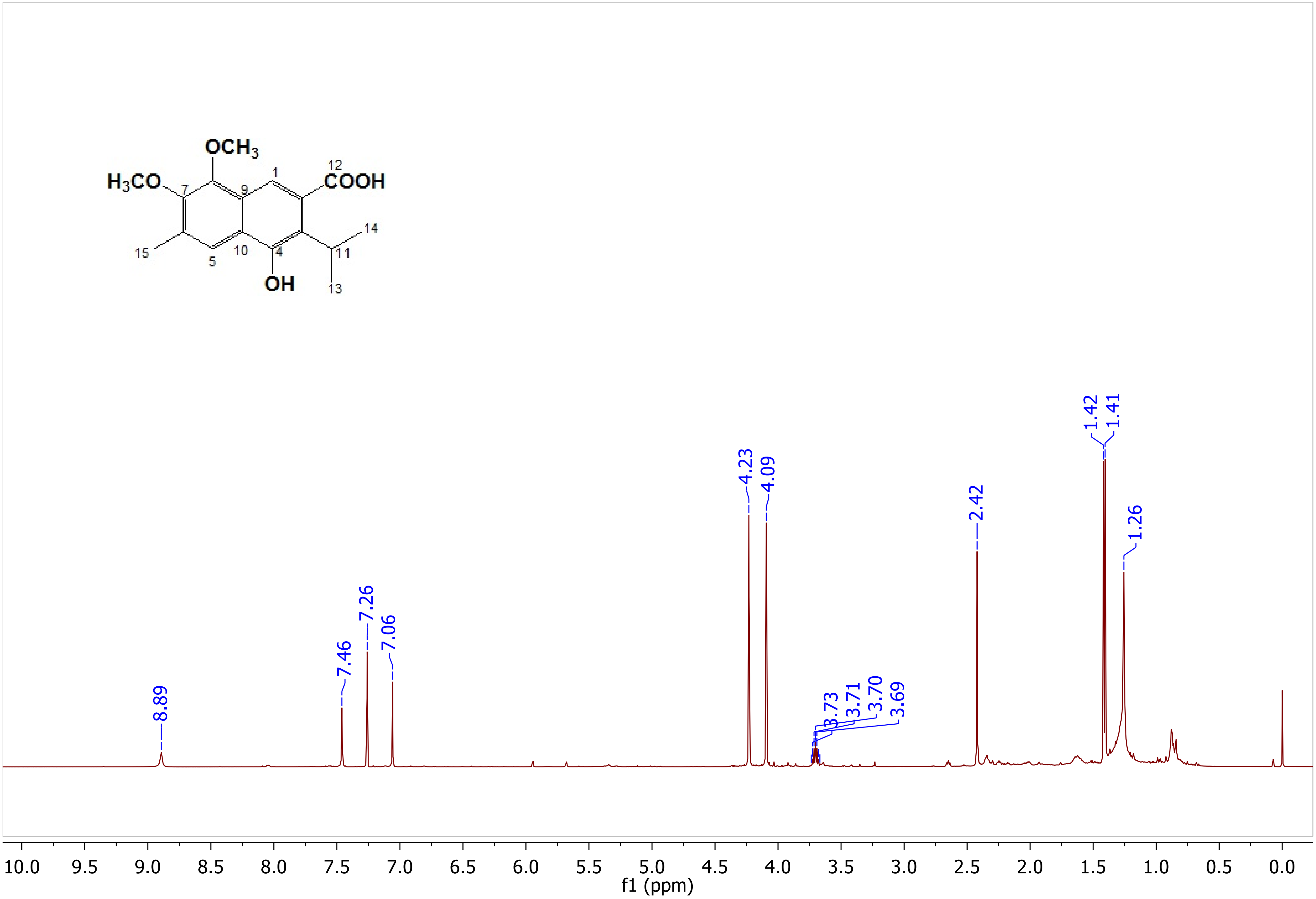

Figure S16. ^{1}H-NMR ($CDCl_3$, 600 MHz) of 8-methyl-iso-sphaeralcic acid (**7**)

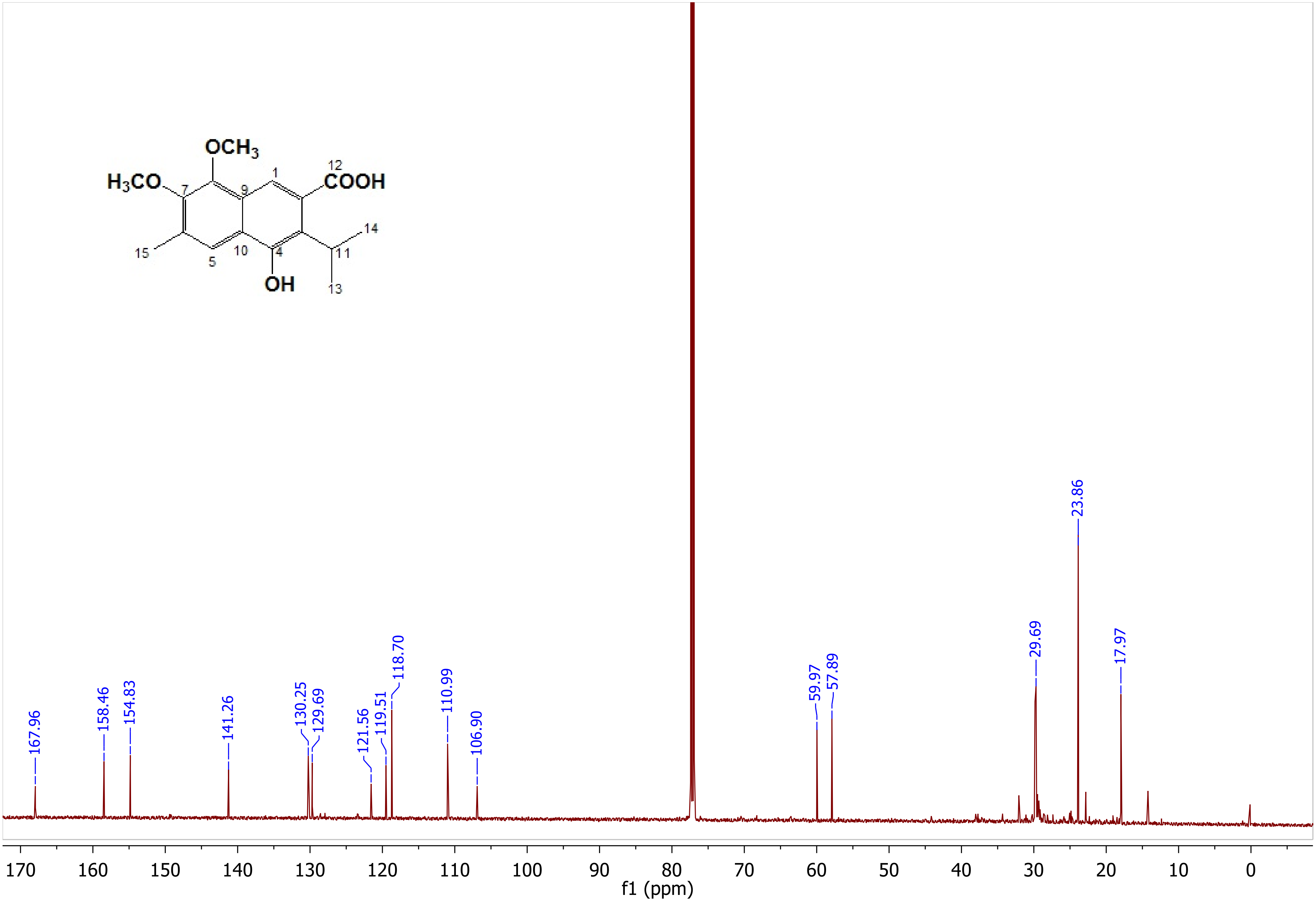

Figure S17. ^{13}C-NMR ($CDCl_3$, 150 MHz) of 8-methyl-iso-sphaeralcic acid (**7**)

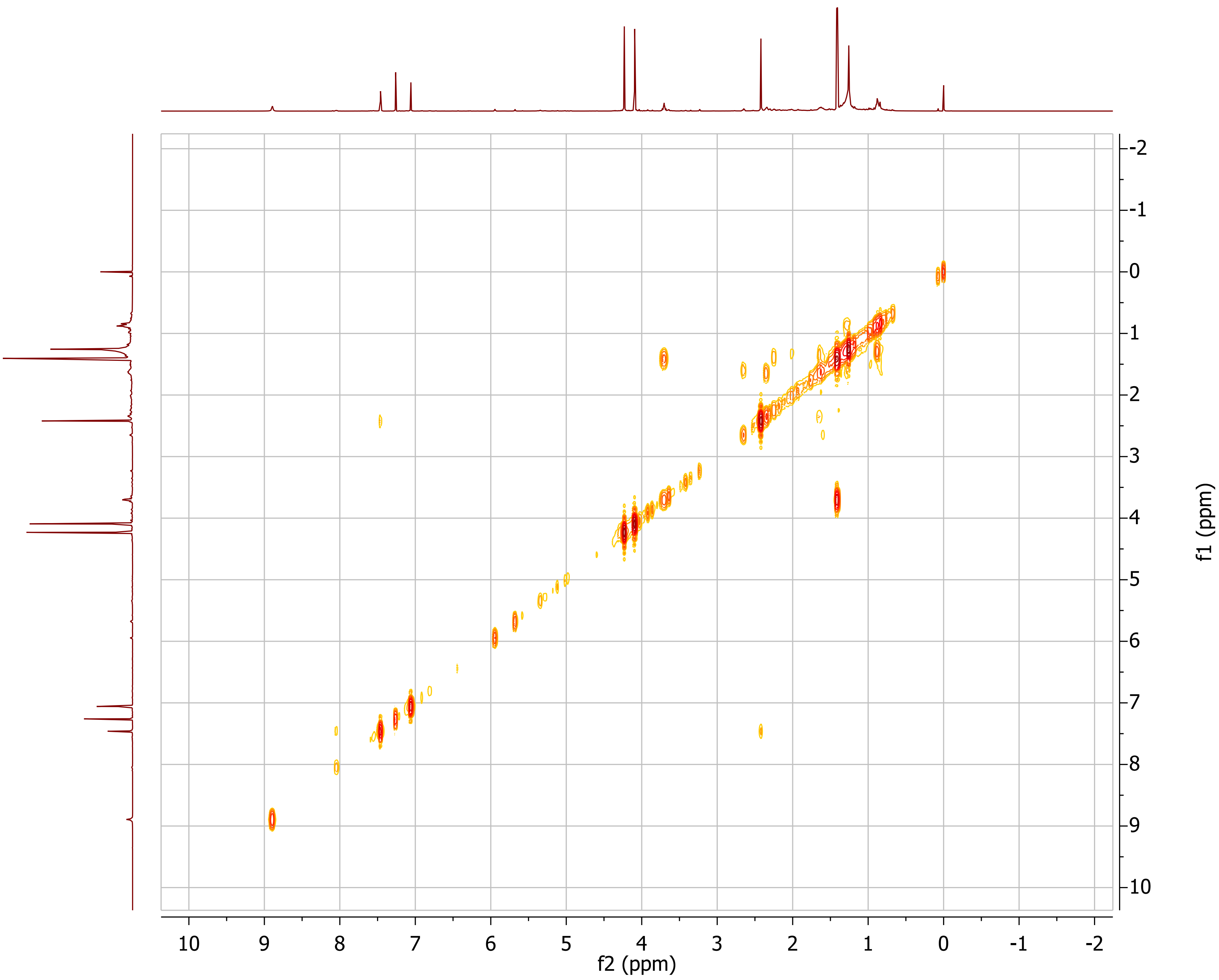

Figure S18. ^1H-^1H (COSY) NMR ($CDCl_3$, 600 MHz) of 8-methyl-iso-sphaeralcic acid (**7**)

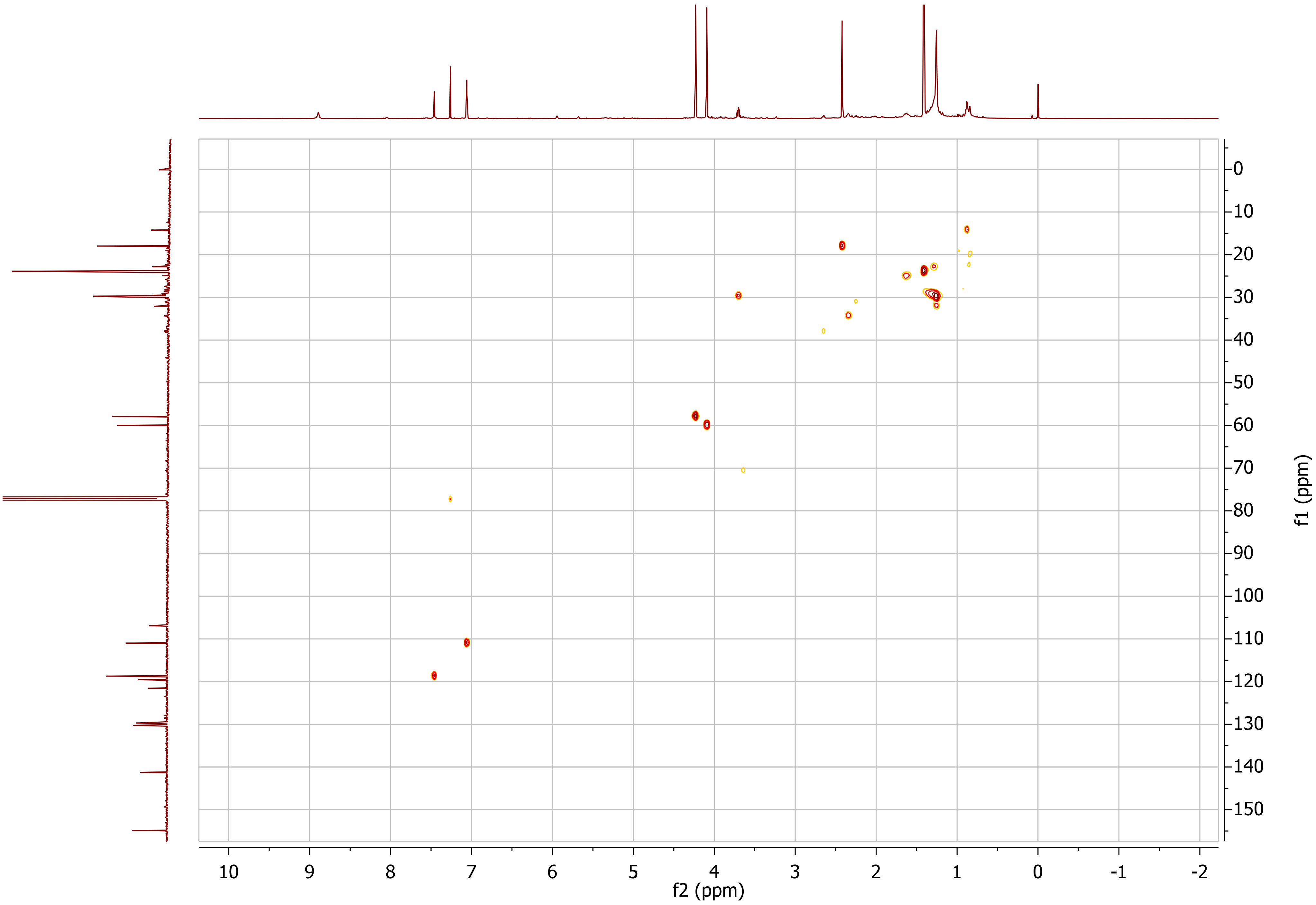

Figure S19. ^{1}H-^{13}C (HSQC) NMR ($CDCl_3$, 600 MHz) of 8-methyl-iso-sphaeralcic acid (**7**)

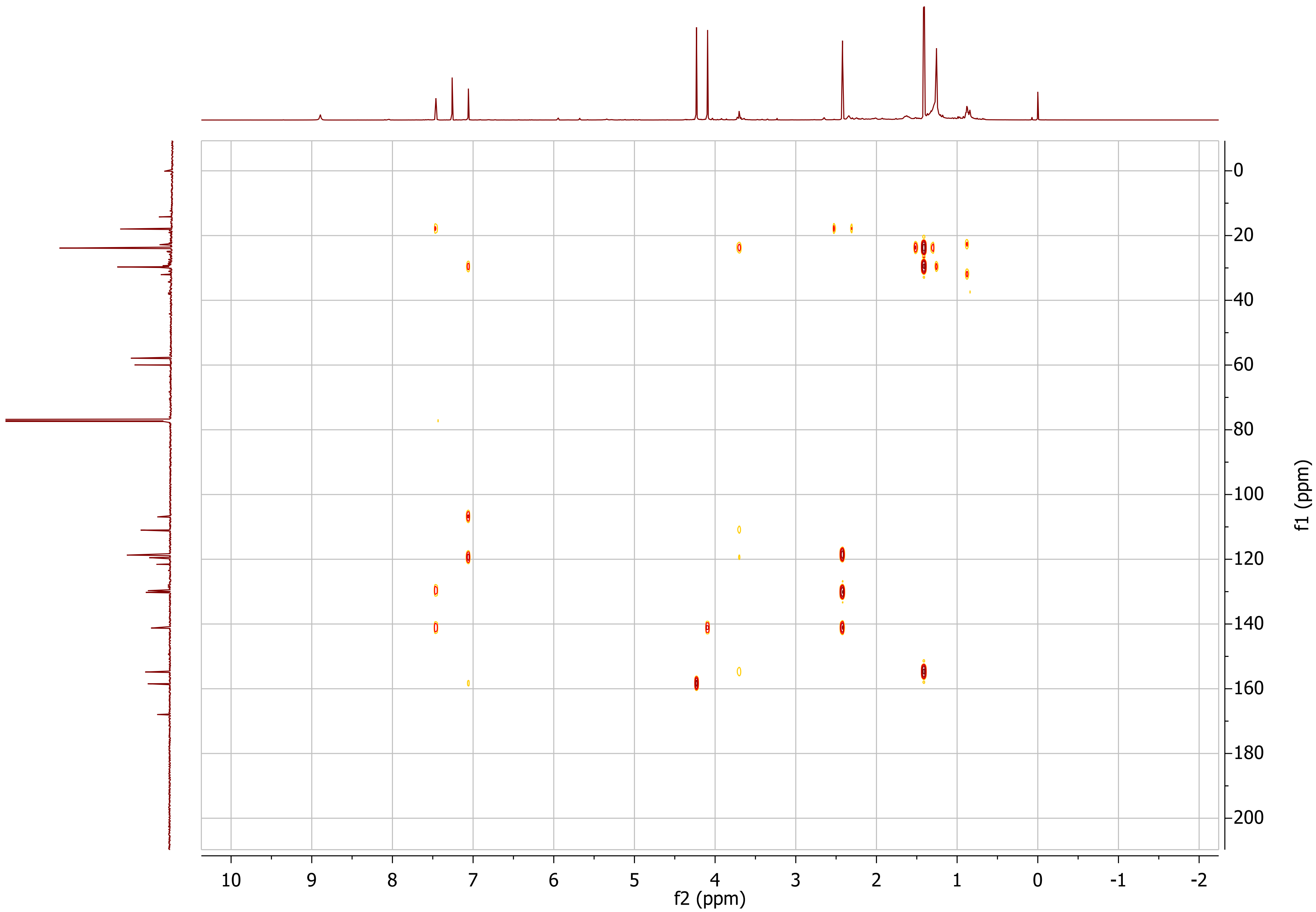

Figure S20. ^{1}H-^{13}C (HMBC) NMR ($CDCl_3$, 600 MHz) of 8-methyl-iso-sphaeralcic acid (**7**)

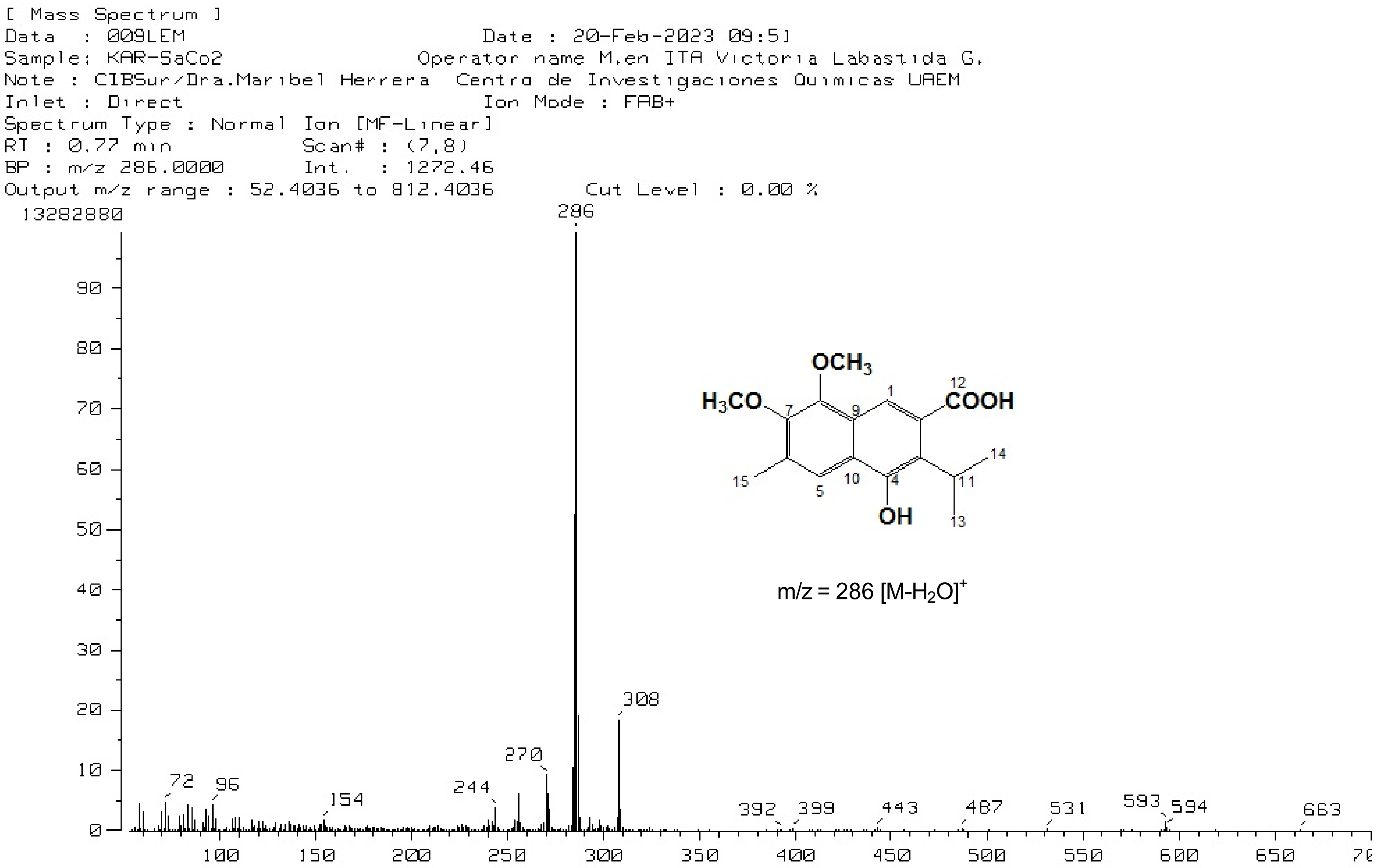

Figure S21. Mass spectrum (FAB-MS) of 8-methyl-iso-sphaeralcic acid (**7**)

Servicio de Espectrometría de Masas
MStation JMS-700 JEOL Alta Resolución
Centro de Investigaciones Químicas UAEM
Tel. 329-79-97 ext.6013

UNIVERSIDAD AUTÓNOMA DEL ESTADO DE MORELOS

CENTRO DE INVESTIGACIONES QUÍMICAS

```
[ Elemental Composition ]
Data  : 009LEM-HR                    Date : 21-Feb-2023 10:36

Sample: KAR-SaCO2               Operator name M.en ITA Victoria Labastida G.
Note : CIBSur/Dra. Maribel Herrera Centro de Investigaciones Químicas UAEM

Inlet : Direct                       Ion Mode : FAB+
RT : 1.15 min                        Scan#: (7,57)

Elements : C 40/0, H 49/0, O 5/0
Mass Tolerance      : 1000ppm, 3mmu if m/z < 3, 25mmu if m/z > 25

Unsaturation (U.S.) : 9.0 - 9.0

Observed m/z Int%   Err[ppm / mmu]    U.S.  Composition
  286.1454   100.0    +87.0 / +24.9     9.0  C 17 H 18 O 4
```

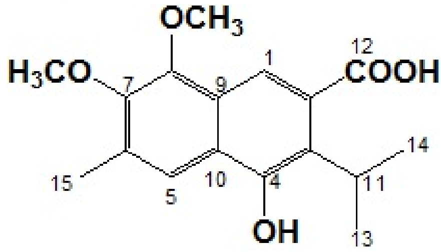

$m/z = 286\ [M-H_2O]^+$
$C_{17}H_{18}O_4$

Figure S22. High resolution mass spectrum (HRFAB-MS) of 8-methyl-iso-sphaeralcic acid (**7**)
